# Aggregation‐Induced Energy Transfer Within a Donor–Acceptor–Donor Compound Featuring Hydrophobic Mesogenic Self‐Assembling Units

**DOI:** 10.1002/asia.70426

**Published:** 2025-11-11

**Authors:** Ryota Usami, Koichiro Ishibashi, Nae Aota, Go Watanabe, Yoshiya Omori, Tsuneaki Sakurai, Masaki Shimizu, Satoshi Minakata, Youhei Takeda

**Affiliations:** ^1^ Department of Applied Chemistry Graduate School of Engineering The University of Osaka Suita Yamadaoka 2‐1, 5650871 Japan; ^2^ Department of Physics School of Science Kitasato University Sagamihara Kanagawa 2520373 Japan; ^3^ Department of Data Science School of Frontier Engineering Kitasato University Sagamihara Kanagawa 2520373 Japan; ^4^ Faculty of Molecular Chemistry and Engineering Kyoto Institute of Technology Hashikami‐cho, Matsugasaki 6068585 Kyoto Japan

**Keywords:** aggregation, charge‐transfer, donor‐acceptor, energy transfer, luminescence

## Abstract

In this study, we report the design and synthesis of a novel donor–acceptor–donor‐type organic emitter incorporating hydrophobic starburst mesogenic units. We have demonstrated that, in aqueous environments, this compound undergoes aggregation that induces efficient energy transfer within the molecules, resulting in tunable shifts in emission color. Molecular dynamics simulations corroborate the proposed mechanism by revealing the intramolecular assembly behavior. These findings provide valuable insights for the rational design of functional nano‐aggregates targeted for applications in sensing and photonic materials.

## Graphical abstract

Hydrophobic mesogenic self‐assembling units induce the aggregation of a structurally distinct dibenzophenazine‐cored electronic donor–acceptor–donor organic emitter in aqueous environments. This aggregation promotes efficient intra‐aggregate energy transfer and results in a drastic shift in emission color.

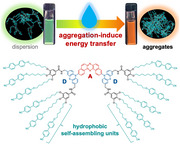

## 1 Introduction

Organic π‐conjugated compounds have attracted considerable attention in materials science due to their unique electro‐ and photo‐active properties arising from extended π‐electron systems [1, 2, 3]. In particular, their photo‐active nature enables diverse applications in optoelectronics [4, 5, 6], bioimaging [7], and sensing technologies [8]. Among these, the photophysical properties in the solid state are of increasing relevance, especially for the development of organic light‐emitting diodes (OLEDs) [9]. However, conventional organic fluorophores often suffer from aggregation‐caused quenching (ACQ) in the condensed phase, primarily due to strong intermolecular electronic interactions and/or exciton delocalization. In this context, the concept of aggregation‐induced emission (AIE) [10, 11, 12] has opened new avenues for the utilization of organic fluorophores in solid‐state environments [5]. A fundamental understanding of how molecular aggregation affects luminescence properties is thus essential for enabling controlled emission behavior via rational molecular assembly design [13].

Previously, we developed dibenzo[*a*,*j*]phenazine (DBPHZ)‐cored organic luminophores featuring an electron donor–acceptor–donor (D–A–D) architecture [14], which exhibit a variety of photophysical behaviors, including thermally activated delayed fluorescence (TADF) [15, 16], room‐temperature phosphorescence (RTP) [17], and stimuli‐responsive luminochromism [18]. Notably, the conformational flexibility of these luminophores plays a crucial role in tuning their emission color. By modulating the population of conformers through external stimuli such as anisotropic forces, thermal energy, solvent vapor, or hydrostatic pressure [19], tunable emission characteristics can be achieved. More recently, we introduced six pairs of an amphiphilic self‐assembling unit, triethylene glycol monomethyl ether (TEGM), into the D–A–D scaffold containing phenothiazine (PTZ) donor units [20]. This chemical modification facilitated the dispersion of luminophores in aqueous environments and hydrophilic matrices such as polyvinyl alcohol (PVA). Molecular dynamics (MD) simulations revealed the coexistence of conformers originating from different orientations (pseudo‐axial or pseudo‐equatorial) of the acceptor unit on the boat‐chair PTZ donors in aqueous media. Within rigid PVA matrices, these conformers displayed ratiometric emission changes, attributed to their distinct quenching responses toward water molecules. Given the stochastic nature of hydrogen bonding among TEGM units, water, and the PVA matrix, we hypothesized that replacing the hydrophilic self‐assembling units with hydrophobic counterparts could provide an effective means to modulate aggregation behavior in aqueous environments. Nevertheless, the influence of such hydrophobic self‐assembling units on aggregation and the resulting photophysical properties remains largely unexplored.

**FIGURE 1:**
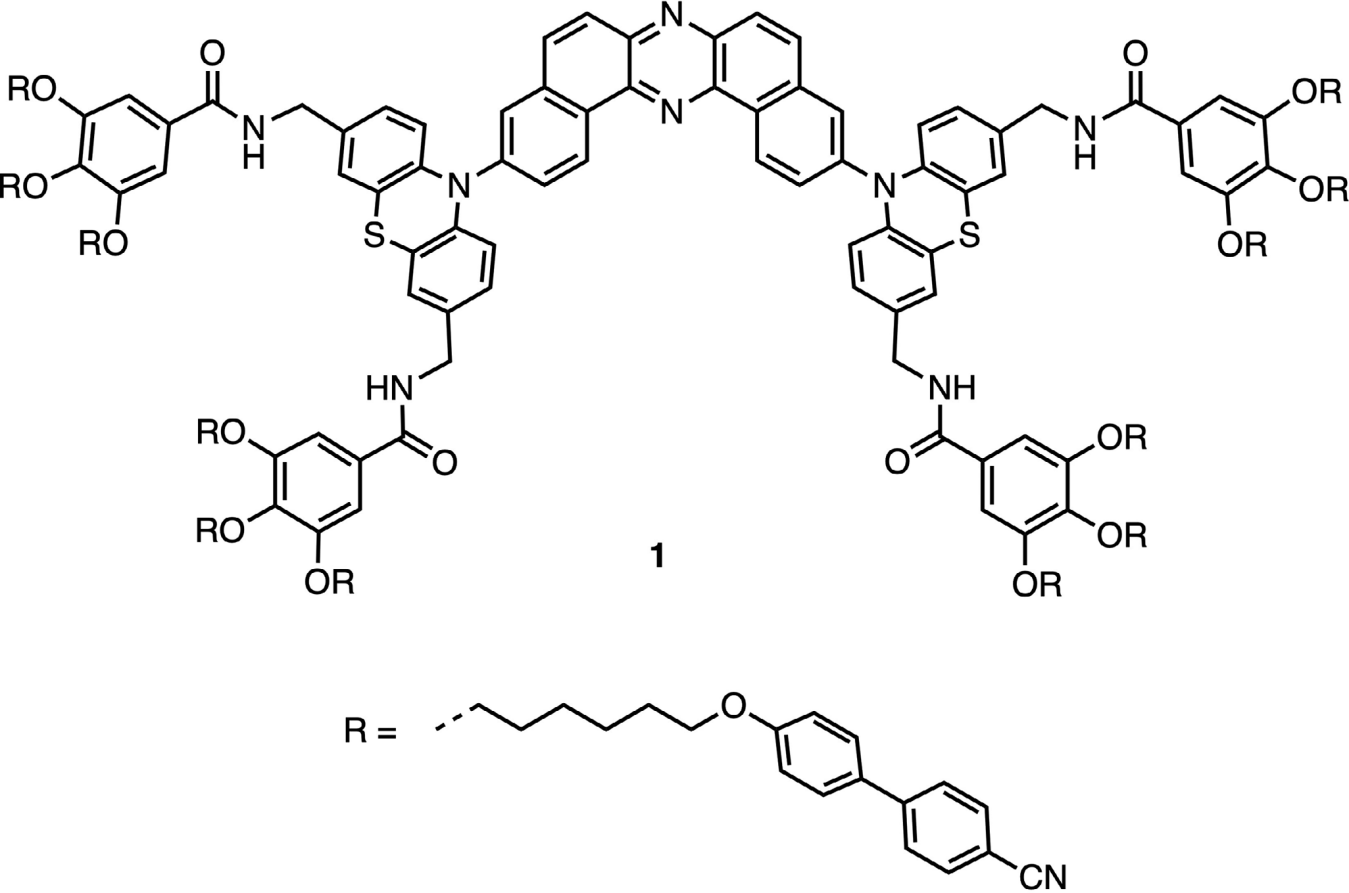
Chemical structure of compound **1**.

Herein, we report the synthesis and aggregation behavior of a novel D–A–D compound, designated as **1**, which incorporates 12 hydrophobic self‐assembling units (Figure 1). In aqueous environments, compound **1** exhibits pronounced AIE behavior. Remarkably, its emission properties are tunable depending on the extent of aggregation, a phenomenon attributed to energy transfer (ET) processes within the aggregate. This tunability highlights the potential of compound **1** as a responsive luminescence material application in aqueous environments.

## 2 Results and Discussion

### 2.1 Design and Synthesis of Materials

As described in the Introduction, we previously developed a water‐dispersible D–A–D compound bearing six hydrophilic units on the PTZ donor moieties [20]. To enhance the self‐assembling capability in aqueous environments, we conceived the strategy of increasing the number of self‐assembling units to elevate the molecular symmetry. As hydrophobic self‐assembling units, we took inspiration from the well‐known nematic liquid crystal compound 4‐cyano‐4’‐pentylbiphenyl (5CB) and designed a D–A–D compound bearing starburst‐type mesogenic groups [21]. Recently, *C*_3_‐symmetry multi‐resonance TADF molecule incorporating the same mesogenic units has been shown to successfully self‐assemble in thin films, enabling controlled molecular orientation for enhanced light out‐coupling [22].

**SCHEME 1:**
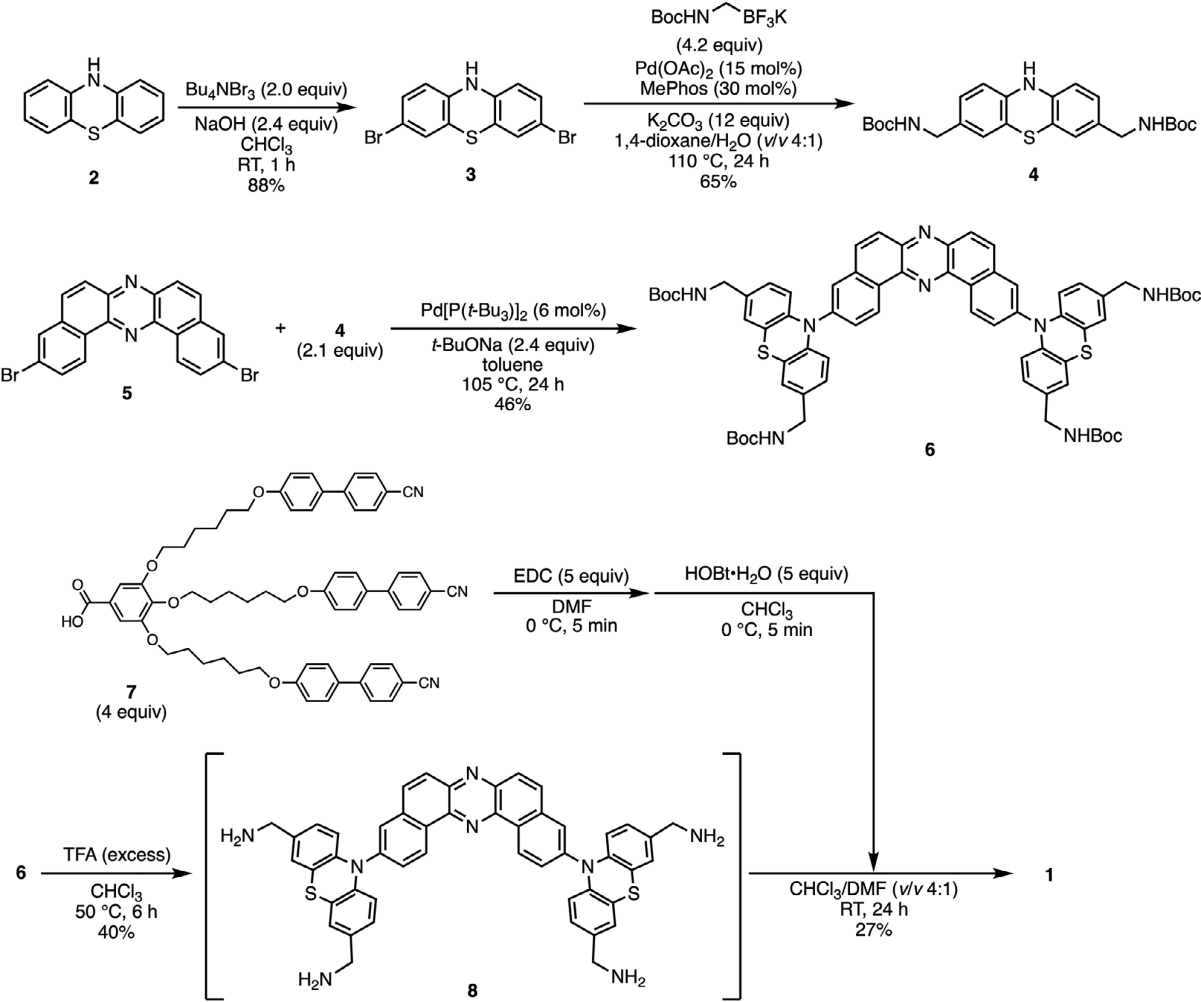
Synthetic route to compound 1.

The designed compound **1** was synthesized following the synthetic route outlined in Scheme 1 (for detailed experimental procedures, see the Supporting Information). Dibromination of phenothiazine (**2**) using tetra‐*n*‐butylammonium tribromide [(*n*‐Bu)_4_NBr_3_] as the brominating agent afforded 3,7‐dibromophenothiazine (**3**) in high yield. A Pd‐catalyzed double Suzuki–Miyaura cross‐coupling reaction between dibromide **3** and *N*‐Boc aminomethyltriflluoroborate [20] afforded bis(*N*‐Boc aminomethyl)phenothiazine **4** in good yield. This electron‐donating unit was subsequently coupled with 3,11‐dibromodibenzophenazine **5** [23] to furnish the D–A–D‐type intermediate **6**. Acidic deprotection of the *N*‐Boc groups yielded intermediate **8**, which bears four aminomethyl substituents. Since the solubility of **8** in organic solvents is quite low, intermediate **8** was subsequently used for the final condensation protocol without isolation (see the Supporting Information for details). To incorporate self‐assembling moieties into the fluorophore, the gallic acid derivative **7** [24] was activated with 1‐ethyl‐3‐(3‐dimethylaminopropyl)carbodiimide (EDC) and 1‐hydroxybenzotriazole (HOBt), and subsequently condensed with **8** to afford the target compound **1**.

### 2.2 Physicochemical and Phase‐Transition Properties of **1**

**FIGURE 2:**
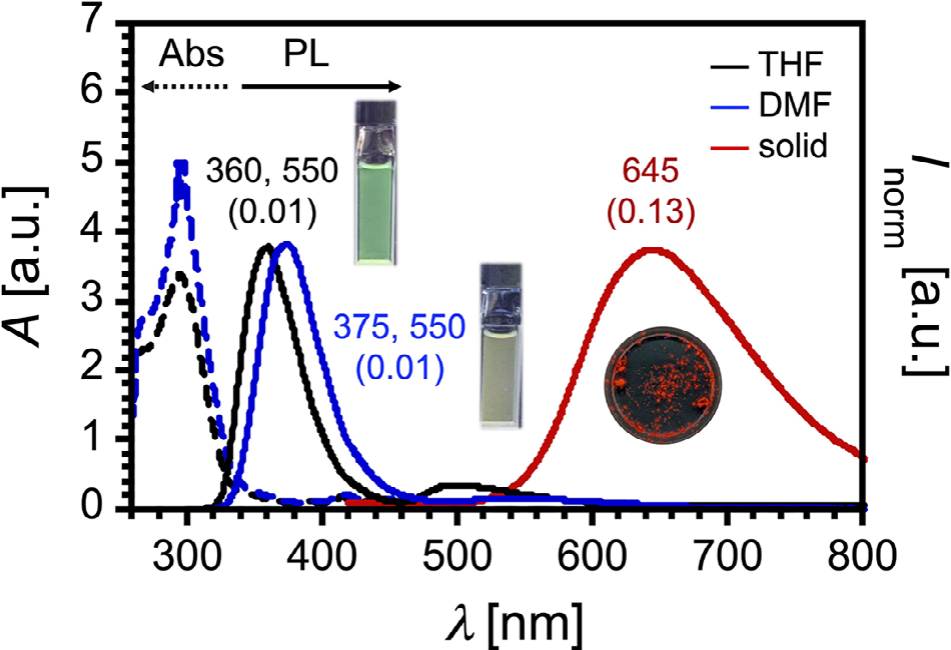
UV‐vis absorption and PL spectra of compound **1** in solutions (*c* ∼ 10^−5^ M) and in the solid state. Black dotted line: UV‐vis absorption in THF; blue dotted line: UV‐vis absorption in DMF; black solid line: PL in THF; blue solid line: PL in DMF; red solid line: PL in the solid state. Excitation wavelength: *λ*_ex_ = 280 nm. Values indicated on the PL spectra (and in parentheses) correspond to the emission maxima (nm) and absolute PLQY, determined using an integrated sphere.

To elucidate the fundamental photophysical characteristics of compound **1**, dilute solutions were prepared in tetrahydrofuran (THF) and *N*,*N*‐dimethylformamide (DMF). UV‐vis absorption and photoluminescence (PL) spectra were subsequently recorded (Figure 2). The UV‐vis absorption spectra of both solutions exhibited comparable profiles and absorption maxima, consistent with the photophysical properties of the D–A–D fluorophore we previously reported [18]. Notably, absorption bands attributed to charge‐transfer (CT) transition were observed in the 450–500 nm range. Upon excitation with UV light (*λ*_ex_ = 280 nm), both solutions displayed weak dual emissions with peaks at 360–375  and 550 nm (Figure 2). This emission behavior contrasts with that of conventional D–A–D compounds, which typically exhibit dominant CT‐type emission in polar solvents. The emission band around 360 nm is attributed to the monomer emission of cyanobiphenylene unit [25], which is reasonable given the presence of twelve such units in comparison to the single D–A–D core.

Interestingly, solidification of the solutions by evaporating solvents resulted in a pronounced red‐shifted emission centered at *λ*_em_ = 645 nm, accompanied by an enhanced photoluminescence quantum yield (PLQY) of 0.13 (Figure 2). This aggregation‐induced enhanced emission (AIEE) behavior is in stark contrast to that observed for previously reported D–A–D molecules [15, 18]. Accordingly, we further investigated the influence of molecular aggregation on the photophysical properties of compound **1**.

**FIGURE 3:**
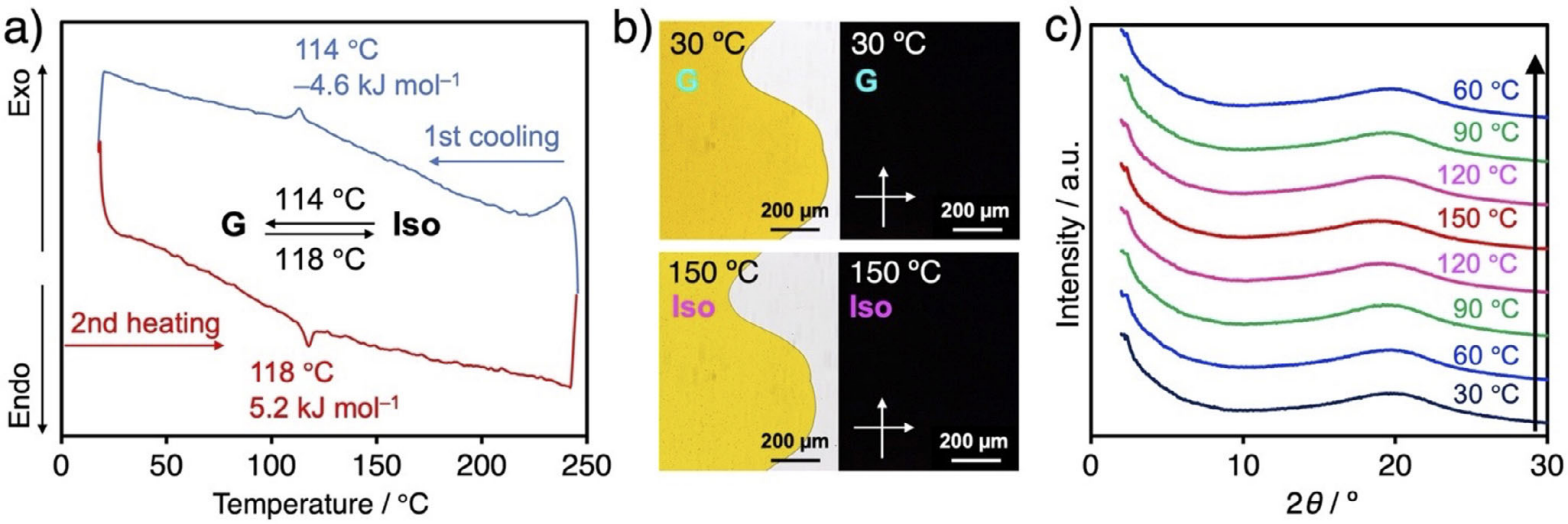
(a) DSC thermogram of compound 1 at 10 °C min^−1^. (b) Optical (left) and crossed polarized optical (right) images of compound **1** at 30 °C (top) and 150 °C (bottom). (c) PXRD patterns of compound **1** at different temperatures. Incident X‐ray wavelength: *λ* = 1.54 Å. G and Iso represent glassy solid and isotropic liquid, respectively.

Since the compound **1** was expected to exhibit liquid‐phase behavior, its phase‐transition properties were investigated by differential scanning calorimetry (DSC), polarized optical microscopy (POM), and powder x‐ray diffraction (PXRD). Upon heating on a hot stage, the initial powdered sample of compound **1** melted at approximately 130 °C, affording an orange‐colored oily liquid. Upon cooling, the liquid solidified without a change in color, accompanied by a gradual decrease in fluidity. DSC analysis revealed a distinct endothermic peak at 118 °C on heating, with an associated enthalpy change of 5.2 kJ mol^−1^. Upon cooling, an exothermic peak was observed at 114 °C, with a corresponding enthalpy change of 4.6 kJ mol^−1^ (Figure 3a). These results demonstrate a single, reversible phase transition in this temperature range. POM observations of compound **1** loaded into a glass cell showed no birefringence at temperatures either above or below the phase transition (Figure 3b), suggesting the absence of long‐range periodic molecular orders. Furthermore, temperature‐dependent PXRD patterns recorded from 30 °C to 150 °C exhibited only broad, featureless halos (Figure 3c). Taking all these data into account and contrary to our initial expectations, we concluded that compound **1** undergoes a single, reversible phase transition between a glassy solid and an isotropic liquid throughout the examined temperature range. Multiple intermolecular and intramolecular interactions involving π–π, hydrophobic, and hydrogen bonding ones may facilitate the non‐periodic aggregation of compound **1**.

**FIGURE 4:**
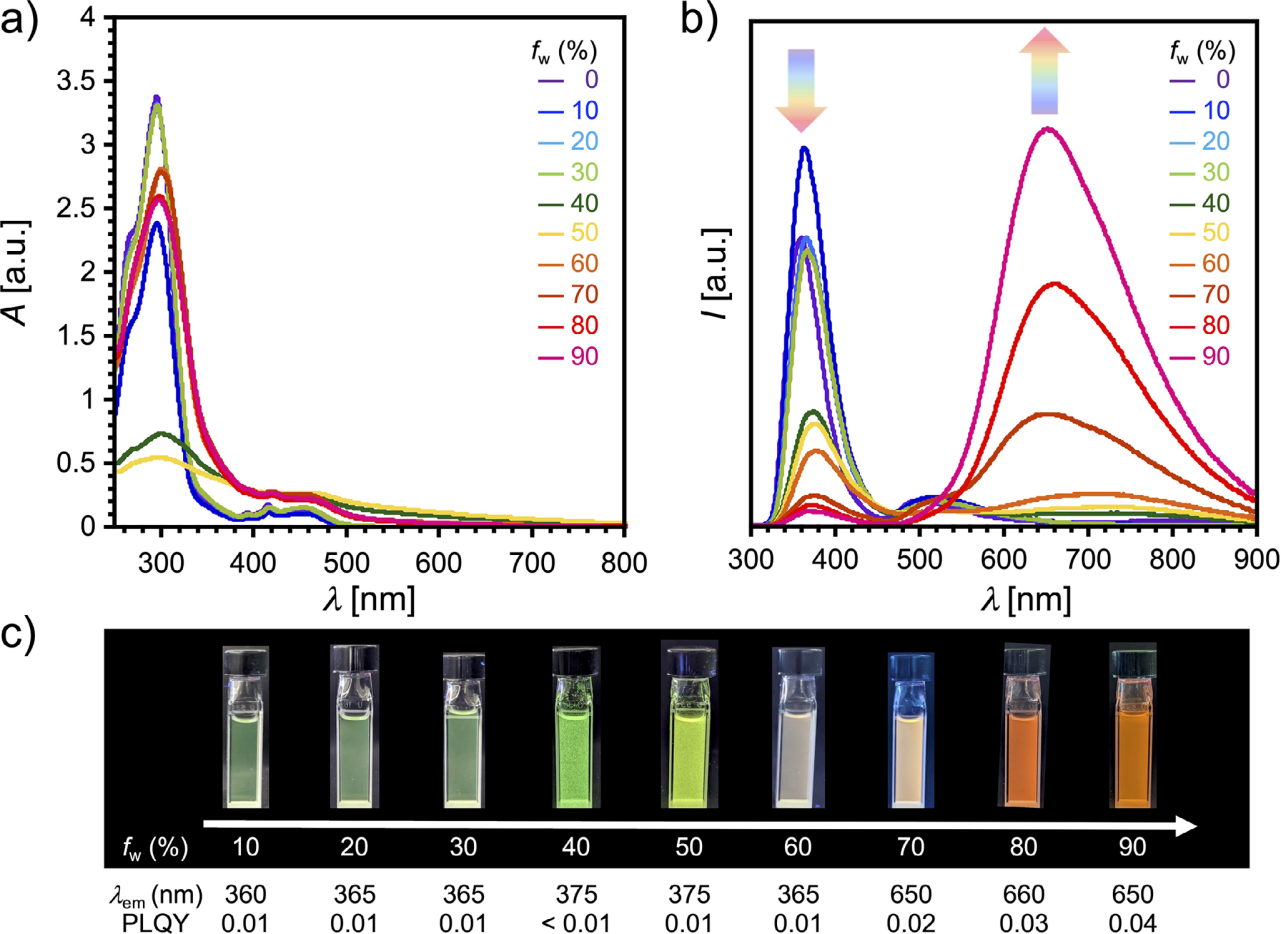
(a) UV‐vis absorption and (b) PL spectra of compound **1** in water/THF mixtures (*c* ∼ 10^−5^ M) with varying water volume fractions (*f*_w_) from 0% to 90%. Excitation wavelength: *λ*_ex_ = 280 nm. (c) Photographs of compound **1** in water/THF mixtures under UV light irradiation (*λ*_ex_ = 365 nm).

Figure 4a,b show the UV‐vis and PL spectra of compound **1** in water/THF mixtures with varying water volume fraction (*f*_w_) ranging from 0% to 90%, respectively. The UV‐vis spectra exhibited stepwise changes depending on the *f*_w_ value (Figure 4a). In the *f*_w_ range of 0%–30%, the spectra remained largely unchanged. However, substantial spectral changes were observed between *f*_w_ = 40%–50%, where the absorption around 300 nm decreased, and a broad tail beyond 500 nm appeared, indicative of the onset of aggregation. From *f*_w_ = 60% to 90%, the spectra evolved further: the absorption around 300 nm partially recovered, while the long‐wavelength tail above 500 nm persisted. These observations suggest that distinct aggregation states form depending on the *f*_w_ value.

The PL spectra showed even more pronounced changes (Figure 4b). In the *f*_w_ range of 0%–30%, the emission centered at *λ*_em_ = 360 nm gradually decreased in intensity. Starting from *f*_w_ = 40%, a new emission band around *λ*_em_ = 700 nm progressively emerged, coinciding with a reduction in the short‐wavelength emission, resulting in dual‐emission behavior (Figure 4b). Above *f*_w_ = 60%, the red‐shifted emission became dominant as *f*_w_ increased. This gradual, ratiometric evolution in the PL profile as a function of *f*_w_ gives rise to a tunable emission color in the water/THF mixture, ranging from green to yellow, whitish orange, and ultimately bright orange (Figure 4c). The PLQY also increased with *f*_w_ above 60%, consistent with typical AIEE behavior.

Our previous work demonstrated that D–A–D fluorophores adopt flexible conformations and exists as a mixture of axial‐axial (ax‐ax), equatorial‐equatorial (eq‐eq), and axial‐equatorial (ax‐eq) conformers in the aggregated state in water [20]. In that case, dual emission was observed, arising from distinct conformers. In contrast, in the present study, only two emission bands were observed: one originating from the cyanobiphenylene moiety [22], and the other attributable to the eq‐eq or eq‐ax conformer of the D–A–D core. This observation implies the occurrence of ET from the high‐energy emissive cyanobiphenylene units to the lower‐energy D–A–D conformers, following the energy hierarchy of eq‐eq < eq‐ax < ax‐ax [26].

Analysis of the UV‐vis absorption spectrum of the aggregated state of compound **1**, in comparison with the PL spectrum of a cyanobiphenyl compound [22], revealed substantial spectral overlap, suggesting the possibility of Förster resonance energy transfer (FRET). Such aggregation‐induced ET systems showing dual emission have been reported [27]. To further investigate this hypothesis, control experiments were performed by mixing a D–A–D compound **9** [18] and a cyanobiphenylene compound **10** (**9**:**10** = 1:4 molar ratio) in water/THF mixtures (Figure S1). As the *f*_w_ increased, the absorption spectrum of the **9**/**10** mixture shifted markedly in a manner similar to that of compound **1**, indicating that aggregation occurs at high *f*_w_ (Figure S1a). In contrast, the PL spectrum of the mixture exhibited a decrease in emission intensity at *λ*_em_ = 370 nm (attributed to compound **10**) as *f*_w_ increased up to 80% (Figure S1b). Moreover, no discernible emission appeared in the orange‐to‐red region (∼650 nm), unlike the strong 650 nm emission observed for compound **1** (Figure S1b *vs*. Figure 4b). Nevertheless, at *f*_w_ = 90%, the **9**/**10** mixture displayed a weak but broad emission centered at 650 nm (Figure S1b), implying that interrmolecular FRET can occur in the mixture and possibly within the aggregates of compound **1** as well.

To further elucidate the origin of aggregation‐induced emissions in the 500–600 nm region, we performed *f*_w_‐dependent emission decay and excitation spectrum measurements for compound **1** (Figures S2 and S3, Tables S1 and S2). Time‐correlated single photon counting (TCSPC) results revealed that the emission lifetime (*τ*) of the 370 nm component gradually increased as *f*_w_ rose from 0% to 60%, then decreased when *f*_w_ was further increased to 90% (Figure S2 and Table S1). In contrast, the *τ* of the 530 nm component increased monotonically with *f*_w_ (Figure S2b and Table S2). The excitation spectrum corresponding to the 370 nm emission in pure THF (*f*_w_ = 0%) exhibited vibronic structure characteristic of the cyanobiphenylene unit. As *f*_w_ increased to 30%, this structure broadened and its intensity decreased with further water addition (Figure S3a), reflecting the strong aggregation tendency of the cyanobiphenylene units. In contrast, the excitation spectra for emissions at 530  and 650 nm retained features characteristics of the D–A–D fluorophore core (Figure S3b,c), confirming that the newly emerging emissions in the 500–700 nm region originate from this core. The differences in emission wavelengths are consistent with previously reported conformation‐dependent emissions [18]. Taken together, these findings indicate the presence of multiple emission pathways for compound **1** in water/THF mixtures depending on the aggregation state. At low *f*_w_ (0%–30%), cyanobiphenylene units begin to aggregate, leading to exciton delocalization along these units and a corresponding increase in *τ* for the 370 nm emission. At intermediate *f*_w_ (40%–70%), long‐lived excitons on the cyanobiphenylene units can transfer energy to the D–A–D core through intra‐ and/or intermolecular FRET pathways.

### 2.3 MD Simulation of Aggregation Formation of **1** in Water

**FIGURE 5:**
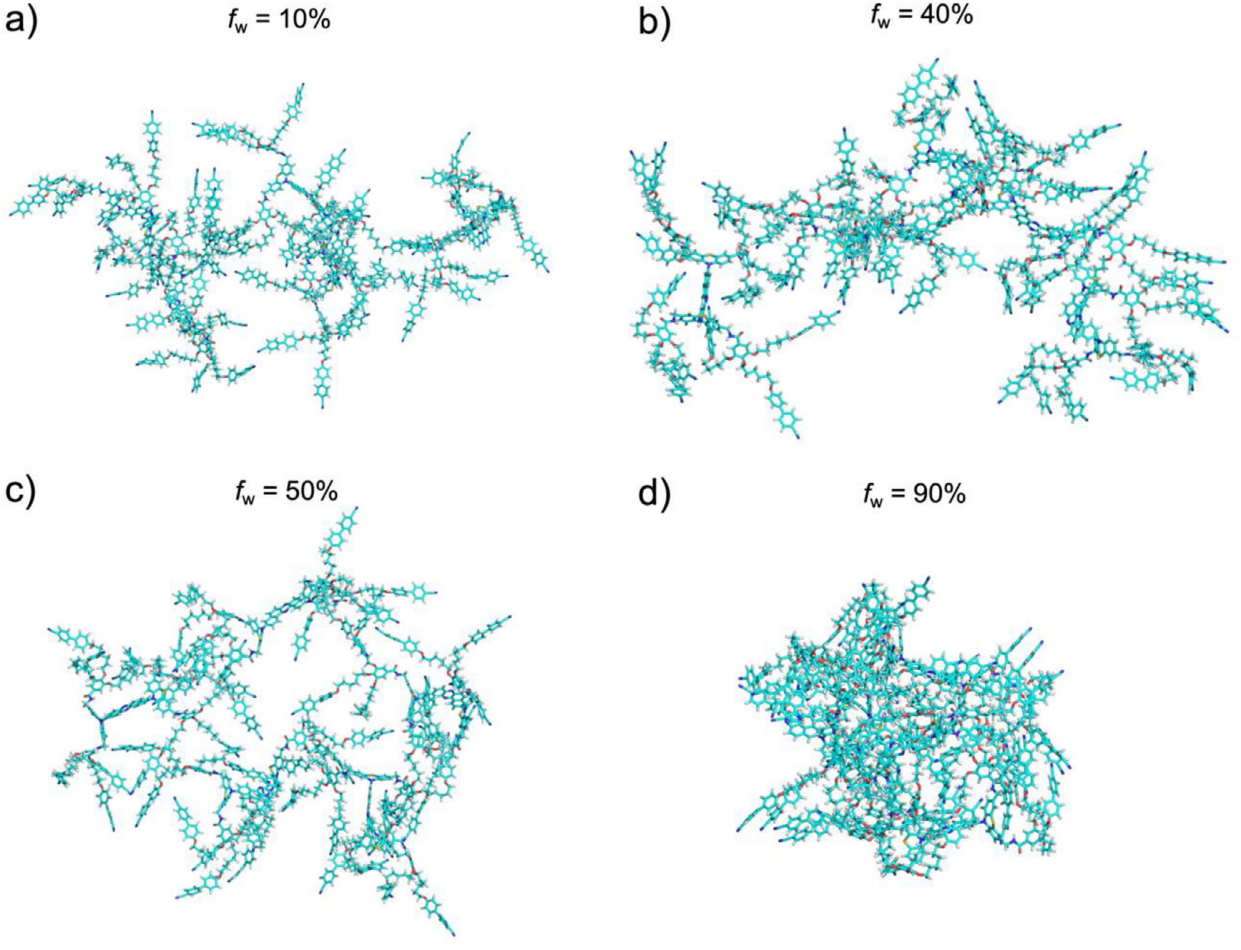
Snapshots from MD simulation of compound **1** in the water/THF mixtures at 200 ns. The systems correspond to (a) *f*_w_ = 10%, (b) *f*_w_ = 40%, (c) *f*_w_ = 50%, and (d) *f*_w_ = 90%. Solvents molecules are omitted for clarity.

To understand the microscopic aggregation behavior of D–A–D compound **1** in water/THF mixtures, we performed MD simulations at various water volume fractions (*f*_w_ = 10%, 40%, 50%, and 90%, Figure 5). In the initial structure for each simulation system, five molecules of compound were randomly distributed in the solvent.

The simulations revealed a strong dependence of the aggregation state on *f*_w_. At a low water content (*f*_w_ = 10%), the molecules formed weak and transient aggregates characterized by intermittent dissociation and weak stacking of the cyanobiphenylene side chains (Figure 5a). At intermediate water fractions (*f*_w_ = 40% and 50%), stable but extended aggregates were formed, with infrequent π‐stacking between the core or side‐chain units (Figure 5b,c). In contrast, at high water content (*f*_w_ = 90%), the molecules coalesced into a single, dense, and spherical cluster (Figure 5d). A key structural feature of this cluster was that the central D–A–D units were shielded from direct contact with each other, being encapsulated by the cyanobiphenylene moieties. The compactness of the aggregates was quantified by the interatomic distance between the most distant atoms within the cluster at 200 ns, which was 6.43 nm for *f*_w_ = 90%, markedly smaller than for *f*_w_ = 40% (11.7 nm) and *f*_w_ = 50% (10.9 nm). These simulation results are in excellent agreement with experimental observations.

**FIGURE 6:**
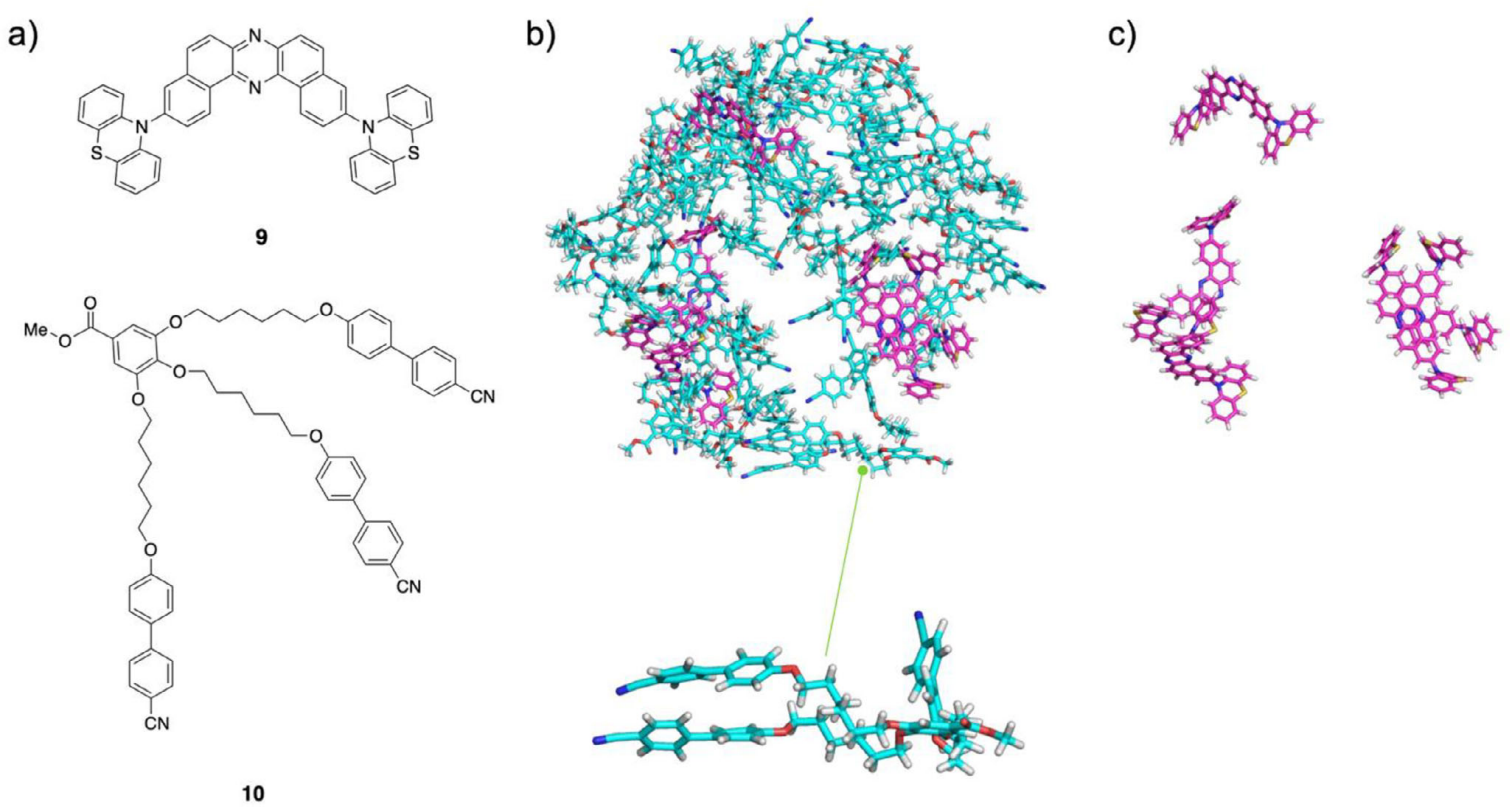
(a) Chemical structures of compounds **9** and **10**. MD simulation snapshots at 200 ns: (b) aggregation formed by compounds **9** and **10** in a water/THF mixture (*f*_w_ = 90%) and (c) molecules of compound **9** extracted from the aggregation. Molecules of compounds **9** and **10** are shown in magenta and cyan, respectively. The enlarged view highlights a representative molecule of compound **10**.

To identify the primary driving force for aggregation, we simulated two model compounds corresponding to the central D–A–D moiety (compound **9**) and the cyanobiphenylene side chain (compound 10) at *f*_w_ = 90% (Figure 6). The cyanobiphenylene molecules readily formed a spherical aggregate, mimicking the behavior of the parent compound **1**. Conversely, the π‐core molecules remained largely dispersed. This clearly demonstrates that the hydrophobic interactions among the cyanobiphenylene moieties are the dominant force driving the self‐assembly. This finding corroborates that, at *f*_w_ = 90%, the D‐A‐D units of compound **1** were surrounded by their cyanobiphenylene moieties, which would induce FRET from the cyanobiphenylene energy donor to the D–A–D energy acceptor.

## 3 Conclusion

In summary, we have developed a new organic emitter based on a donor–acceptor–donor architecture incorporating multiple starburst‐type hydrophobic mesogenic self‐assembling units. Although the compound did not exhibit liquid crystalline behavior, it displayed a unique aggregation‐induced ET phenomenon in aqueous environments. Molecular dynamics simulations supported the intramolecular organization of the hydrophobic units around the central fluorophore, facilitating efficient ET. This study opens a new avenue for the development of nano‐ordered aggregate materials with tunable emission colors dependent on the degree of aggregation, potential for innovative sensing systems.

## Conflicts of Interest

The authors declare no conflicts of interest.

## Supporting information




**Supporting Information file 1**: asia70426‐sup‐0001‐SuppMat.pdf

## Data Availability

The data that supports the findings of this study are available in the supplementary material of this article
