# Supplementary material for: Aggregation‐Induced Energy Transfer Within a Donor–Acceptor–Donor Compound Featuring Hydrophobic Mesogenic Self‐Assembling Units
Source: Chem Asian J. 2025 Nov 11;21(1):e70426. doi: 10.1002/asia.70426 (PMC12802537; doi:10.1002/asia.70426)
Supplement: Supplementary file 1 — Supporting Information file 1: asia70426‐sup‐0001‐SuppMat.pdf [file ASIA-21-e70426-s001.pdf]

Supporting Information for

Aggregation-Induced Energy Transfer within Donor–Acceptor–Donor Compound Featuring Hydrophobic Mesogenic Self-Assembling Units

Ryota Usami,[a] Koichiro Ishibashi,[b] Nae Aota,[a] Go Watanabe,*[b, c] Yoshiya Omori,[d] Tsuneaki Sakurai,[d] Masaki Shimizu,[d] Satoshi Minakata,[a] and Youhei Takeda*[a]

[a] *Department of Applied Chemistry, Graduate School of Engineering, The University of Osaka Yamadaoka 2-1, Suita 5650871, Japan*

[b] *Department of Physics, School of Science, Kitasato University, Sagamihara, Kanagawa 252-0373, Japan*

[c] *Department of Data Science, School of Frontier Engineering, Kitasato University, Sagamihara, Kanagawa 252-0373, Japan*

[d] *Faculty of Molecular Chemistry and Engineering, Kyoto Institute of Technology, Hashikami-cho, Matsugasaki, Sakyo-ku, Kyoto 6068585, Japan*

E-mail: go0325@kitasato-u.ac.jp; takeda@chem.eng.osaka-u.ac.jp

Table of Content

**General Remarks**	S1
**Synthetic Procedures and Spectroscopic Data of New Compounds**	S2–S6
**Control Experiment (Figure S1)**	S7
**Emission Decay Profiles of Compound 1 in a water/THF mixture with varied f_w (Figure S2 and Table 1 and S2)**	S7–S9
**Excited Spectra of Compound 1 in a water/THF mixture with varied f_w (Figure S3)**	S9
**MD Simulations (Table S3 and S4)**	S9–S10
**Copies of NMR Charts of New Compounds**	S11–14
**References**	S15

General Remarks. All reactions were carried out under an atmosphere of nitrogen unless otherwise noted. Products were purified by chromatography on silica gel Chromatorex BW-300 and NH-DM1020 (Fuji Silysia Chemical Ltd.). Analytical thin-layer chromatography (TLC) was performed on pre-coated silica gel glass plates (Merck silica gel 60 F_{254} TLC plate and Fuji Silysia Chromatorex NH, 0.25 mm thickness). Compounds were visualized with UV lamp. Melting points were determined on a Stanford Research Systems MPA100 OptiMelt Automated Melting Point System. All ^{1}H and ^{13}C NMR were recorded on a JEOL JMTC-400/54/SS Spectrometer (^{1}H NMR, 400 MHz; ^{13}C NMR, 100 MHz) using tetramethylsilane as an internal standard. Infrared spectra were acquired on a SHIMADZU IRAffinity-1 FT-IR Spectrometer. Mass spectra and High-resolution mass spectra were obtained on a JEOL JMS-700 Mass Spectrometer. UV-Vis spectra were recorded on a Shimadzu UV-2550 spectrophotometer. All the steady-state UV-Vis absorption and PL spectra were measured at room temperature with diluted solutions (10^{-5} M), which were prepared from degassed spectroscopic grade solvents (N_2 for 30 min). Steady-state emission spectra were recorded on a JASCO FP-8650 NIR spectrofluorometer, and absolute photoluminescence quantum yields were calculated with a HAMAMATSU Quantaurus-QY C11347-01 spectrometer with an integrating sphere. Lifetime measurement was conducted with a time-correlated single-photon counting (TCSPC) system HAMAMATSU Quantaurus-Tau C11367. Polarized optical microscopy (POM) images were recorded on an Olympus BX53-P polarizing optical microscope equipped with a Mettler HS82 hot-stage system. Differential scanning calorimetry (DSC) was performed on a Hitachi High-Tech Science Corporation DSC620 differential scanning calorimeter. Powder X-ray diffraction (PXRD) patterns were recorded on a Rigaku MiniFlex600 X-ray diffractometer (λ = 1.54 Å) with a D/teX Ultra semiconductor detector. The sample was mounted on a silicon non-reflecting plate.

Materials. Dehydrated toluene and THF used for organic synthesis were purified by passing through a solvent purification system. 3,11-dibromo-dibenzo[*a*,*j*]phenazine (**5**) [CAS No. 1620543-64-7],[S1] 3,4,5-tris((6-((4'-cyano-[1,1'-biphenyl]-4-yl)oxy)hexyl)oxy)benzoic acid (**7**),[S2] 3,11-di(10*H*-phenothiazin-10-yl)dibenzo[*a*,*j*]phenazine (**9**),[S3] and methyl 3,4,5-tris((6-((4'-cyano-[1,1'-biphenyl]-4-yl)oxy)hexyl)oxy)benzoate (**10**)[S2] were synthesized according to the reported procedure. Other reagents were purchased and used as received. Solvents of fluorescence spectroscopic grade for measurement of UV-Vis and emission spectra were purchased from Nacalai Tesque Inc.

Synthetic Procedures and Spectroscopic Data of New Compounds

*Synthesis of 3,7-dibromo-10H-phenothiazine (**8**) [CAS No. 21667-32-3]*

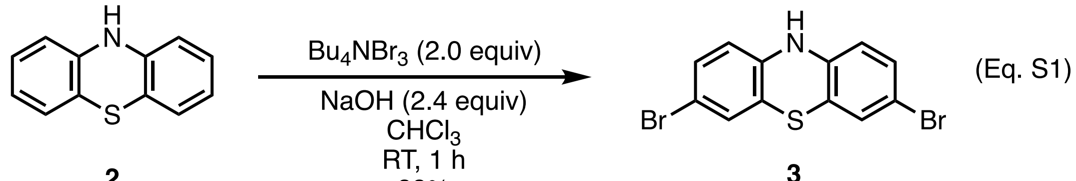

2 (996.5 mg, 5.0 mmol) **3** (1.57 g, 4.4 mmol)

To a three-necked flask (300 mL) equipped with a magnetic stir bar, were added phenothiazine (**2**) (996.5 mg, 5.0 mmol) and $(n\text{-Bu})_4NBr_3$ (4.85 g, 10 mmol, 2.0 equiv), and NaOH (488.0 mg, 12.2 mmol, 2.4 equiv). The flask was sealed with rubber septa, evacuated under reduced pressure, and purged with nitrogen gas. This cycle was repeated for 3 times. A 200 mL of $CHCl_3$ was injected through septum, and the resulting reaction mixture was stirred at room temperature for 1 h. The reaction was quenched with sat. aqueous sodium thiosulfate (200 mL), and organic layer was extracted with $CHCl_3$ (100 mL×3). The combined organic layer was dried over Na_2SO_4, filtered, and solvents of filtrate were evaporated under reduced pressure to give crude product, which was purified by flash column chromatography on NH silica gel (eluent: *n*-hexane/EtOAc = 1:1). The collected fractions were combined, and the solvents were evaporated under reduced pressure to give the title compound **3** including small amounts of impurities. The solid was reprecipitated from *n*-hexane/EtOA, which was then collected by filtration. The solid was dried under vacuum to provide the title compound **3** as pale green solid in 88% yield (1.57 g, 4.4 mmol). Mp 191–193 °C; R_f 0.22 (*n*-hexane/EtOAc 4:1, NH-silica gel); ^{1}H NMR (400 MHz, DMSO-d_6): δ 8.85 (s, 1H), 7.14–7.11 (m, 4H), 6.57 (d, J = 8.0 Hz, 2H); ^{13}C NMR (100 MHz, DMSO-d_6): δ 140.9, 130.3, 128.1, 118.2, 116.0, 112.7; IR (ATR): ν 3317, 3072, 3051, 1869, 1587, 1452, 1384, 1286, 1236, 1136, 1089, 1080, 933, 881, 858, 808, 750, 729 cm^{-1}; HRMS (FAB$^+$, NBA): *m*/*z* calcd for $C_{12}H_7Br_2NS$ 354.8666, found 354.8673. These spectroscopic data were in good agreement with that previously reported in literature.[S4]

*Synthesis of di-tert-butyl ((10H-phenothiazine-3,7-diyl) bis (methylene)) dicarbamate (**4**)*

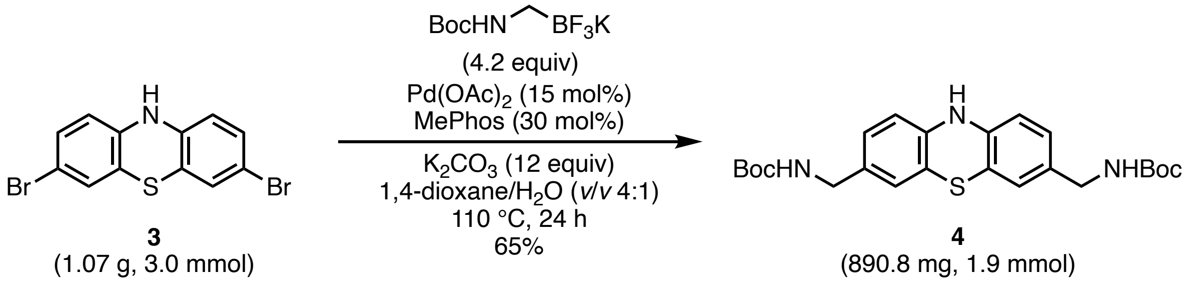

(Eq. S2)

To a two-necked flask (100 mL) equipped with a magnetic stirring bar, were added 3,7-dibromophenothiazine (**3**) (1.07 g, 3.0 mmol), potassium *N*-Boc aminomethyl trifluoroborate (2.99 g, 12.6 mmol, 4.2 equiv), $Pd(OAc)_2$ (101.5 mg, 0.45 mmol, 15 mol%), MePhos (328.4 mg, 0.90 mmol, 30 mol%), and K_2CO_3 (4.98 g, 36 mmol, 12 equiv). The flask was sealed with rubber septa, evacuated under reduced pressure, and purged with nitrogen gas. This cycle was repeated for 3 times. 1,4-dioxane (19.2 mL) and water (4.8 mL) were injected into the flask through a septum, and the resulting mixture was stirred under reflux condition at 110 °C for 24 h. The reaction mixture was allowed to cool to room temperature, and sat. aqueous $NaHCO_3$ (20 mL) was added. The organic layer was extracted with $CHCl_3$ (20 mL×3), and combined organic layer was dried over Na_2SO_4 and filtered. Solvents in filtrate were evaporated under reduced pressure to give crude product, which was purified by flash chromatography on NH silica gel (eluent: *n*-hexane/EtOAc = 4:1 to 1:1). The collected fractions were combined, and the solvents were evaporated under reduced pressure to give the title compound **4** including small amounts of impurities. The solid was reprecipitated from *n*-hexane/EtOA, which was then collected by filtration. The solid was dried under vacuum to provide the title compound **4** as white solid in 65% yield (890.8 mg, 1.9 mmol). Mp 183–185 °C; R_f 0.29 (*n*-hexane/EtOAc 1:1, NH-silica gel); ^{1}H NMR (400 MHz, DMSO-d_6): δ 8.49 (s, 1H), 7.26 (t, J = 6.0 Hz, 2H), 6.82 (d, J = 8.0 Hz, 2H), 6.75 (s, 2H), 6.58 (d, J = 8.4 Hz, 2H), 3.90 (d, J = 6.4 Hz, 4H), 1.36 (s, 18H); ^{13}C NMR (100 MHz, DMSO-d_6): δ 155.7, 140.7, 133.5, 126.4, 124.9, 115.8, 114.1, 77.7, 42.6, 28.2; IR (ATR): ν 3383, 3280, 3008, 2981, 2926, 2891, 2875, 2831, 2818, 2806, 1683, 1517, 1475, 1454, 1431, 1413, 1390, 1365, 1298, 1284, 1267, 1236, 1190, 1168, 1116, 1091, 1043, 1026, 941, 881, 860, 813 cm^{-1}; HRMS (FAB$^+$, NBA): *m*/*z* calcd for $C_{24}H_{31}N_3O_4S$ 457.2035, found 457.2018.

*Synthesis of tetra-tert-butyl ((dibenzo[a,j]phenazine-3,11-diylbis(10H-phenothiazine-10,3,7-triyl))tetrakis(methylene))tetracarbamate (**6**)*

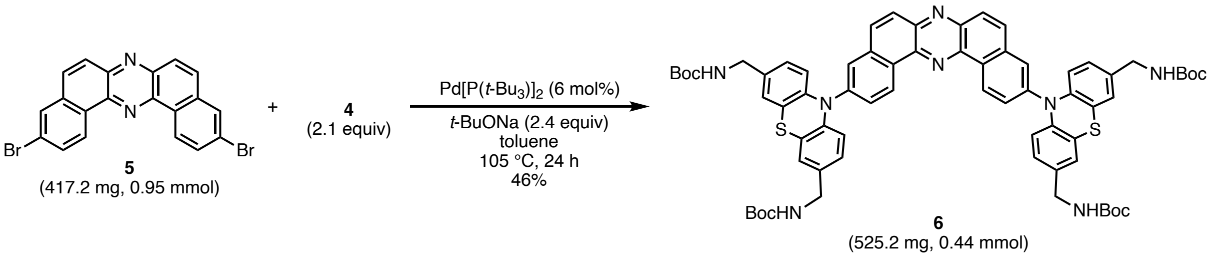

(Eq. S3)

To a two-necked reaction tube (50 mL) equipped with a magnetic stirring bar, were added 3,11-dibromo-dibenzo[*a,j*]phenazine (**5**) (417.2 mg, 0.95 mmol), aminomethylated phenothiazine **4** (916.0 mg, 2.0 mmol, 2.1 equiv), $Pd[P(t\text{-}Bu_3)]_2$ (26.2 mg, 0.048 mmol, 6.0 mol%), and *t*-BuONa (223.9 mg, 2.3 mmol, 2.4 equiv). The tube was sealed with rubber septa, evacuated under reduced pressure, and purged with nitrogen gas. This cycle was repeated for 3 times. Toluene (5.8 mL) was injected to the tube through a septum, and the resulting mixture was stirred at 105 °C for 24 h. The reaction mixture was allowed to cool to room temperature. Water (20 mL) was added to the mixture, and organic layer was extracted with $CHCl_3$ (20 mL×3), and combined organic layer was dried over Na_2SO_4 and filtered. Solvents were evaporated from the filtrate under reduced pressure to give crude product, which was purified by flash column chromatography on NH silica gel (eluent: *n*-hexane/EtOAc = 4:1 to 1:1). The collected fractions were combined, and the solvents were evaporated under reduced pressure to give the title compound **6** including small amounts of impurities. The solid was reprecipitated from *n*-hexane/$CHCl_3$, which was then collected by filtration. The solid was dried under vacuum to provide the title compound **6** as yellow-orange solid in 46% yield (525.2 mg, 044 mmol). R_f 0.14 (*n*-hexane/ EtOAc 1:1, NH-silica gel); ^{1}H NMR (400 MHz, $CDCl_3$): δ 9.71 (d, J = 8.4 Hz, 2H), 8.10 (d, J = 8.8 Hz, 2H), 8.04 (d, J = 9.2 Hz, 2H), 7.90 (s, 2H), 7.77 (d, J = 8.8 Hz, 2H), 7.06 (s, 4H), 6.84 (d, J = 7.6 Hz, 4H), 6.44 (d, J = 8.0 Hz, 4H), 4.84 (brs, 4H), 4.18 (d, J = 4.8 Hz, 8H), 1.44 (s, 36H); ^{13}C NMR (100 MHz, $CDCl_3$): δ 155.7, 142.8, 142.78, 142.71, 140.3, 135.2, 134.2, 132.0, 129.6, 127.8, 127.7, 127.6, 126.4, 126.2, 126.2, 123.0, 118.2, 79.5, 43.6, 28.3; IR (ATR): ν 3350, 2974, 2928, 1684, 1611, 1599, 1503, 1474, 1429, 1389, 1364, 1356, 1246, 1163, 1070, 1047, 1028, 997, 972, 937, 858, 793, 783, 766, 721 cm^{-1}; HRMS (FAB$^+$, NBA): m/z calcd for $C_{68}H_{70}N_8O_8S_2$ 1190.4758, found 1190.4753.

*Synthesis of D–A–D compound **1***

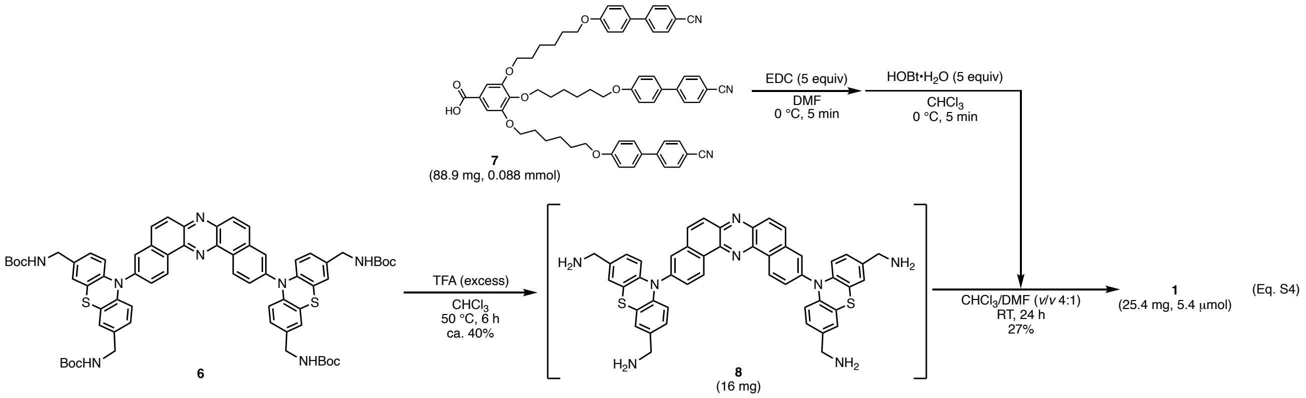

*Step 1: Preparation of tetra-tert-butyl (dibenzo[a,j]phenazine-3,11-diylbis(10H-phenothiazine-10,3,7-triyl))tetramethanamine (**8**)*: To a two-necked flask (100 mL) equipped with a magnetic stirring bar, was added compound **6** (240.4 mg, 0.20 mmol). The flask was sealed with rubber septa, evacuated under reduced pressure, and purged with nitrogen gas. This cycle was repeated for 3 times. Chloroform (40 mL) was injected to the flask through a septum. To the resulting solution, trifluoroacetic acid (8.6 mL, 112 mmol, 520 equiv) was added under a stream of N_2 gas, and the resulting mixture stirred at 50 °C for 6 h. The reaction mixture was allowed to cool to room temperature. NaOH aq. (2.0 M) was added to the mixture to neutralize the reaction mixture, and organic layer was extracted with $CHCl_3$ (20 mL×3). The combined organic layer was dried over Na_2SO_4 and filtered. Solvents were evaporated from the filtrate under reduced pressure to give crude product. Solid was reprecipitated from *n*-hexane/$CHCl_3$, which was then collected by filtration. The solid was dried under vacuum to provide the title compound **8** as yellow solid in ca. 40% yield (62.6 mg, 0079 mmol). Due to the difficulty in purifying the compound **8**, the compound was characterized by ^{1}H NMR, IR, and HRMS, and subjected to the following condensation reaction. Mp 125 °C (dec.); R_f 0.27 ($CHCl_3$/MeOH 9:1, NH-silica gel); ^{1}H NMR (400 MHz, $CDCl_3$): δ 9.72 (d, J = 8.8 Hz, 2H), 8.11 (d, J = 9.2 Hz, 2H), 8.05 (d, J = 9.6 Hz, 2H), 7.90 (d, J = 2.0 Hz, 2H), 7.79 (dd, J = 8.4, 2.0 Hz, 2H), 7.16 (d, J = 2.0 Hz, 4H), 6.92 (dd, J = 8.0, 2.0 Hz, 4H), 6.57 (d, J = 8.0 Hz, 4H), 3.77 (brs, 8H); IR (ATR): ν 3628, 3358, 3008, 2997, 2981, 2926, 2891, 2875, 2833, 2818, 2806, 1595, 1544, 1473, 1452, 1354, 1303, 1251, 1192, 1168, 1116, 1101, 1091, 997, 937, 920, 854, 813, 798, 723, 698 cm^{-1}; HRMS (FAB$^+$, NBA): m/z calcd for $C_{48}H_{38}N_8S_2$ 790.2661, found 790.2650.

*Step 2: Condensation of **8** with 3,4,5-tris((6-((4'-cyano-[1,1'-biphenyl]-4-yl)oxy)hexyl)oxy)benzoic acid (7)*: To a two-necked reaction tube (50 mL) equipped with a magnetic stirring bar, was added compound **7** (88.9 mg, 0.088 mmol, 4.0 equiv). The tube was sealed with rubber septa, evacuated under reduced pressure, and purged with nitrogen gas. This cycle was repeated for 3 times. DMF

(3.0 mL) was injected to the tube through a septum. To the resulting solution was stirred at 0 °C for 5 min (*Solution A*).

To another two-necked reaction tube (10 mL) equipped with a magnetic stirring bar, was added 1-hydroxybenzotriazole monohydrate (HOBt•H_2O) (15.9 mg, 0.10 mmol, 5.0 equiv). The tube was sealed with rubber septa, and purged with nitrogen gas. This cycle was repeated for 3 times. $CHCl_3$ (4.0 mL) was injected to the tube through a septum. The resulting solution was injected to *Solution A*, and the resulting mixture was stirred 0 °C for 5 min (*Solution B)*.

To the other two-necked reaction tube (10 mL) equipped with a magnetic stirring bar, was added intermediate **8** (16 mg, ca. 0.020 mmol). The tube was sealed with rubber septa, and purged with nitrogen gas. This cycle was repeated for 3 times. $CHCl_3$ (4.0 mL) was injected to the tube through a septum. The resulting solution was cooled to 0 °C. To this solution, *Solution B* was injected through septum, and the resulting reaction mixture was stirred at room temperature for 24 h. Water (5.0 mL) was added to the mixture, which was washed with HCl aq. (2.0 M, 20 mL×2), NaOH aq. (2.0 M, 30 mL×2), and water (30 mL). The separated organic layer was dried over Na_2SO_4 and filtered. Solvents were evaporated from the filtrate under reduced pressure to give crude product, which was purified by flash column chromatography on NH silica gel (eluent: *n*-hexane/EtOAc = 1:2 to 1:1, and then $CHCl_3$ only). The collected fractions were combined, and the solvents were evaporated under reduced pressure to give the title compound **1** including small amounts of impurities. This was further purified by preparative TLC and reprecipitation from *n*-hexane/$CHCl_3$. Removal of supernatant to dry the solid under reduce pressure gave the title compound **1** as yellow solid in 11% in 2 steps (25.4 mg, 5.4 μmol). R_f 0.23 ($CHCl_3$ only, NH-silica gel); ^{1}H NMR (400 MHz, $CDCl_3$): δ 9.67 (d, J = 8.8 Hz, 2H), 8.08 (d, J = 9.2 Hz, 2H), 7.99 (d, J = 9.2 Hz, 2H), 7.85 (s, 2H), 7.69 (dd, J = 8.4, 1.6 Hz, 2H), 7.64–7.60 (m, 24H), 7.58–7.54 (m, 24H), 7.47–7.44 (m, 24H), 7.12 (s, 4H), 7.01 (s, 8H), 6.93–6.90 (m, 28H), 6.52 (t, J = 5.6 Hz, 4H), 6.40 (d, J = 8.4 Hz, 4H), 4.48 (d, J = 4.4 Hz, 8H), 4.00 (dd, J = 11.2, 6.4 Hz, 24H), 3.95 (t, J = 6.4 Hz, 24H), 1.86–1.72 (m, 48H), 1.59–1.48 (m, 48H); ^{13}C NMR (100 MHz, $CDCl_3$): δ 167.00, 159.64, 159.59, 152.96, 145.05, 145.04, 142.83, 142.77, 142.56, 140.99, 140.28, 135.26, 133.69, 132.80, 132.49, 131.16, 129.71, 129.15, 128.24, 126.93, 126.73, 126.70, 122.84, 119.08, 119.04, 118.07, 114.95, 109.96, 109.89, 105.69, 73.21, 69.06, 67.94, 67.85, 43.14, 30.16, 29.19, 29.17, 29.10, 25.87, 25.83, 25.77, 25.73; IR (ATR): ν 3374, 3040, 2938, 2864, 2224, 1659, 1603, 1580, 1524, 1493, 1474, 1425, 1385, 1354, 1329, 1314, 1292, 1248, 1179, 1113, 1074, 1063, 1031, 1012, 999, 935, 908, 853, 820, 764, 725 cm^{-1}; HRMS (FAB$^+$, NBA): *m*/*z* calcd for $C_{304}H_{282}N_{20}O_{28}S_2$ 4724.1, found 4726.8.

Control Experiment

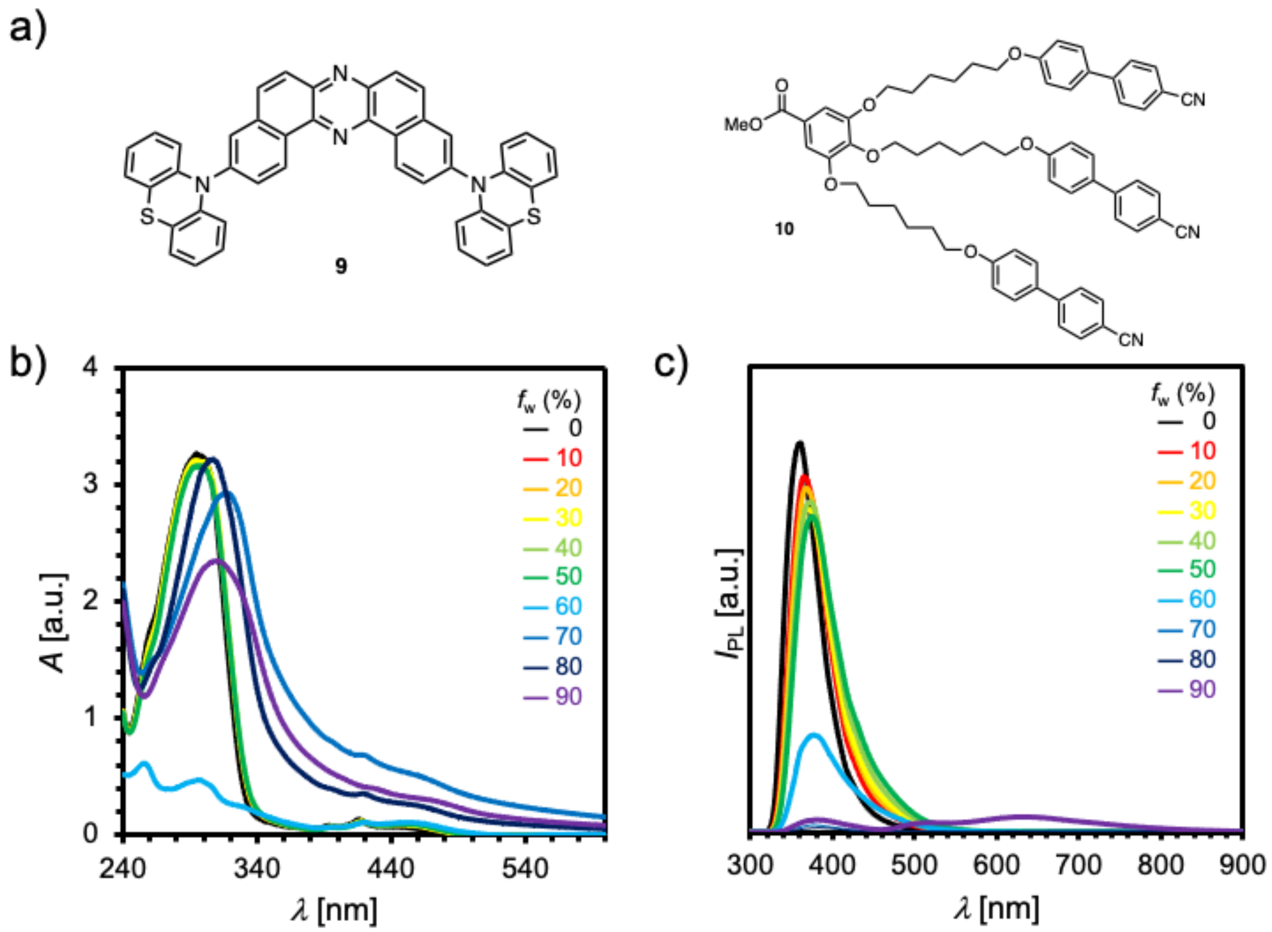

Figure S1. a) Chemical structures of compounds **9** and **10**; b) UV-vis absorption and c) PL spectra of the 1:4 mixture of compounds **9** and **10** in water/THF mixtures (c ~ 10^{-5} M) with varying water volume fractions (f_w) from 0% to 90%. Excitation wavelength: λ_{ex} = 280 nm.

Emission Decay Profiles of Compound 1 in a water/THF mixture with varied f_w

For the TCSPC measurements, PL decay curves were deconvoluted with instrumental responsive function (IRF), and decay function G(t) was fitted with the following equation to extract lifetimes:

$$G(t) = \sum_{i=1}^{n} A_i \exp\left(-\frac{t}{\tau_i}\right)$$

where τ_i are lifetimes of each emission component and A_i are the corresponding fractional amplitudes. Also, intensity average lifetime $<\tau>$ was calculated by the following equation:

$$<\tau> = \frac{\sum_{i=1}^{n} \tau_i^2 A_i}{\sum_{i=1}^{n} \tau_i A_i}$$

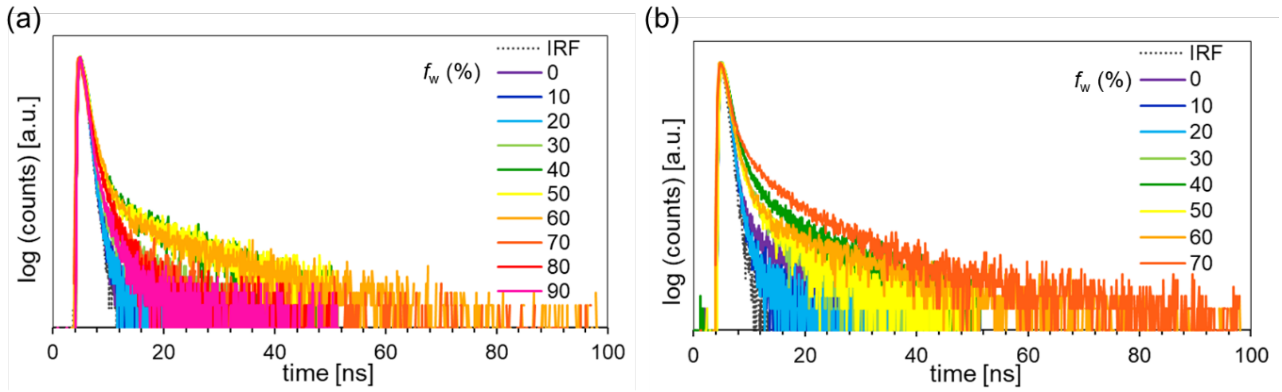

Figure S2. Emission decay profiles of **1** in water/THF mixture ($c \sim 10^{-5}$ M) with varying water volume fractions (f_w). a) Emission decay at λ_{em} = 370 nm; b) λ_{em} = 530 nm. Excitation wavelength: λ_{ex} = 280 nm.

Table S1. Summary of TCSPC measurements of the emission-decay of **1** in water/THF mixture at λ_{em} = 370 nm with varied water-content fraction.[a]

f_w	$<\tau>$	τ_1	τ_2	A_1	A_2	χ^2
0	0.064	0.034	0.77	6577	12.4	1.10
10	0.065	0.032	1.20	6758	5.25	1.25
20	0.061	0.031	0.74	6904	12.8	1.39
30	0.23	0.10	2.43	2546	6.11	1.47
40	9.71[b]	9.71	–	3.78	-	1.18
50	11.1[b]	11.1	–	3.10	-	1.16
60	12.2[b]	12.2	–	4.73	-	0.92
70	3.13[b]	3.13	–	17.5	-	0.94
80	0.36	0.035	1.76	6136	28.9	1.21
90	0.20	0.034	1.97	6432	10.7	1.03

[a] Excited at 280 nm. [b] Life time was determined by tail fitting method.

Table S2. Summary of TCSPC measurements of the emission-decay of **1** in water/THF mixture at λ_{em} = 530 nm with varied water-content fraction.[a]

f_w	$<\tau>$	τ_1	τ_2	A_1	A_2	χ^2
0	5.46[b]	5.46	–	2.15	-	0.92
10	0.20	0.15	1.70	1779	4.64	1.33
20	0.21	0.16	1.52	1708	6.76	1.33
30	4.21[b]	4.21	–	8.98	–	1.10
40	5.43[b]	5.43	–	6.00	–	0.98

50	4.81[b]	4.81	–	6.58	–	1.03
60	6.80[b]	6.80	–	44.0	–	1.21
70	9.17[b]	9.17	–	19.8	–	1.05

[a] Excited at 280 nm. [b] Life time was determined by tail fitting method.

Excited Spectra of Compound 1 in a water/THF mixture with varied f_w

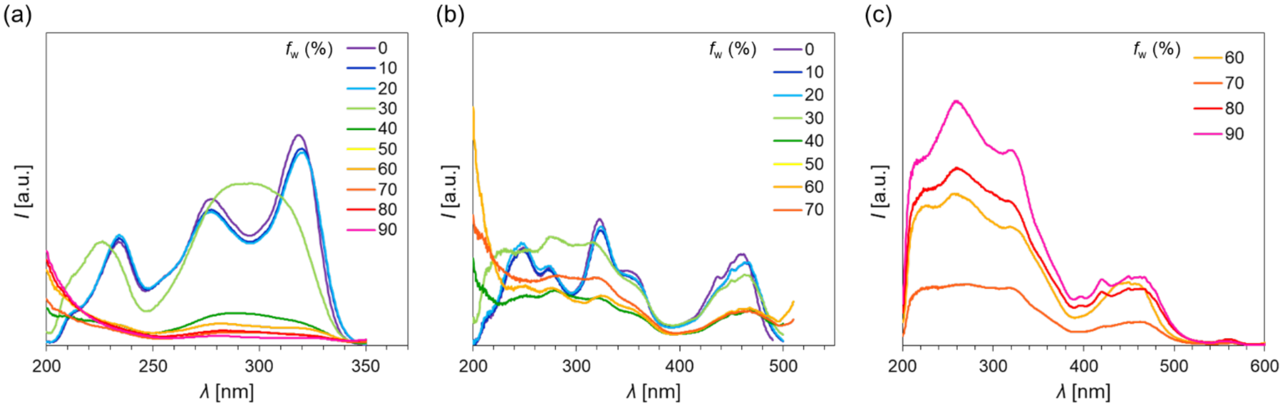

Figure S3. Excitation spectra of **1** in water/THF mixture (c ~ 10^{-5} M) with varying water volume fractions (f_w). a) Excitation spectra of λ_{em} = 370 nm, (b) λ_{em} = 530 nm, and (c) λ_{em} = 650 nm. Excitation wavelength: λ_{ex} = 280 nm.

MD simulations

All-atom MD simulations were performed using the GROMACS 2016.6 package. For the system containing compound **1**, 5 molecules of its equatorial conformer were randomly placed in a MD simulation box. The box was then solvated with a mixture of water and THF molecules at four difference water volume fractions (f_w): 10 %, 40 %, 50 %, and 90 %. The exact number of solvent molecules for each system is detailed in Table S1. The other system was prepared with 5 molecules of compound **9** and 20 molecules of compound **10**. These solutes were placed in a water/THF mixture with f_w = 90 % (see Table S2 for the number of solvent molecules). Similar to compound **1**, the equatorial conformer was used for compound **9**.

The partial atomic charges for compounds **1**, **9**, and **10** and THF were calculated using the restrained electrostatic potential (RESP)[S5] methodology based on DFT calculations (B3LYP/6-31G(d,p)) using the GAUSSIAN 16 revision C01 program package. The generalized Amber force field (GAFF)[S6] parameters were used for compounds **1**, **9**, and **10** and THF. Water molecules were described by the TIP4P-EW model. Several parameters were modified based on previous studies. First, specific proper dihedral angle parameters at the donor-acceptor linkage of compound **1** and **9**

were adjusted to accurately reproduce the potential energy profile of the equatorial conformer.[S7] Furthermore, the van der Waals and specific proper dihedral angle parameters for the cyanobiphenylene moiety were also modified.[S8, S9]

The same simulation protocol was applied to all systems. First, the steepest descent energy minimization was done for each system. This was followed by a 5 ns pre-equilibration run in the NPT ensemble, where the temperature (300 K) and pressure (1 bar) were maintained using the Berendsen thermostat and barostat[S10] with relaxation times of 0.2 ps and 2.0 ps, respectively. Subsequently, a 200 ns equilibration run was performed for each system at 300 K and 1 bar. For these runs, the Nosé-Hoover thermostat[S11] and Parrinello-Rahman barostat[S12] were employed with relaxation times of 1.0 and 5.0 ps, respectively. All bonds involving hydrogen atoms were constrained with the LINCS[S13] algorithm, allowing a time step of 2 fs. The long-range Coulomb interactions were handled using the smooth particle mesh Ewald[S14] method with a grid spacing of 0.30 nm. The real space cut-off of 1.2 nm was applied to both the short-range Coulomb and van der Waals interactions.

Table S3. Composition of the simulation systems containing compound **1** at different water volume fractions (f_w).

f_w	90%	50%	40%	10%
THF	693	3525	4310	6477
water	28402	16022	13061	3270

Table S4. Composition of the simulation system containing compound **9** and **10** at f_w = 90%.

f_w	90%
THF	694
water	28443

Copies of NMR Charts of New Compounds

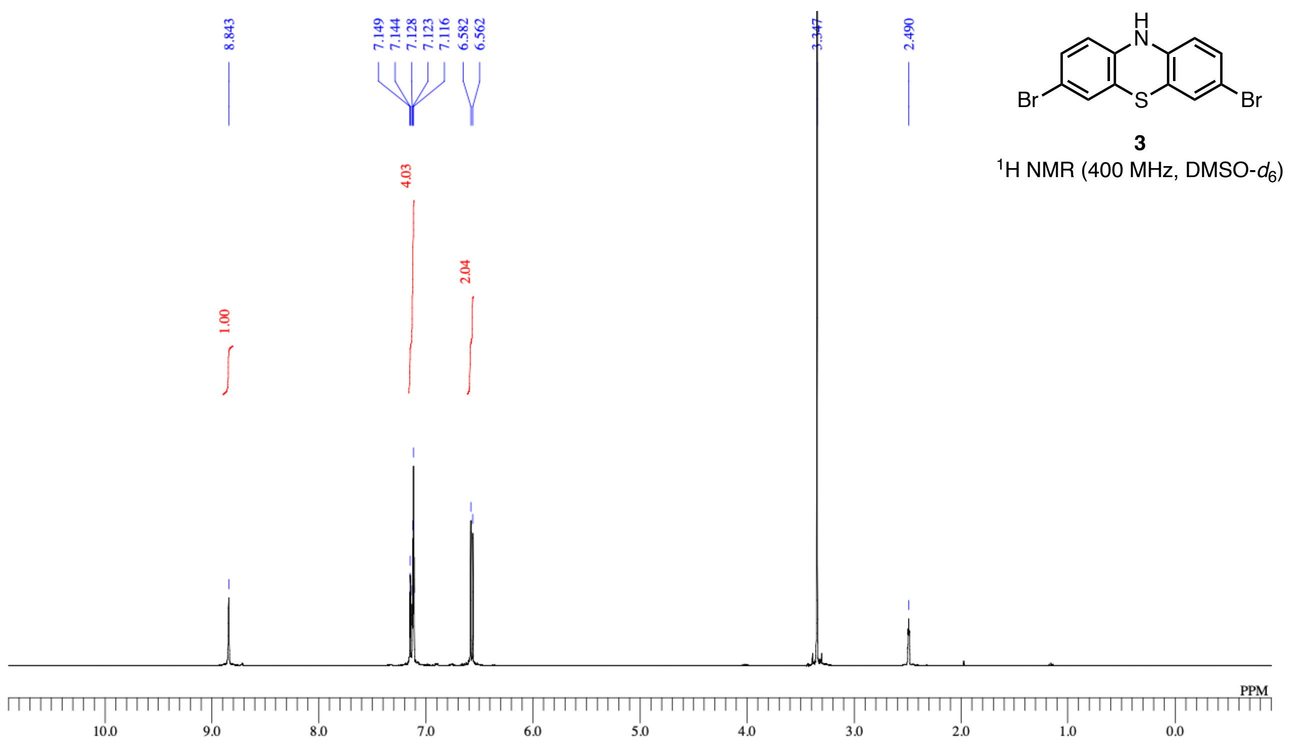

3

^{1}H NMR (400 MHz, DMSO-d_6)

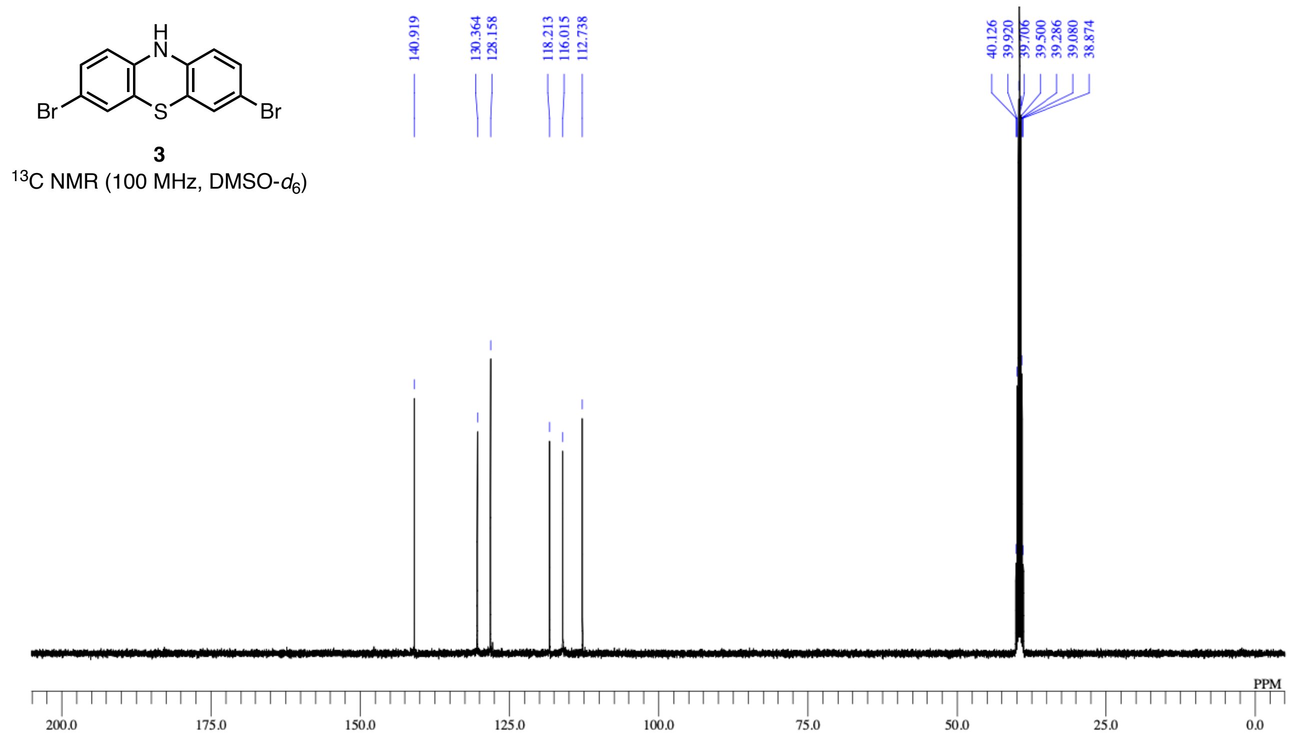

3

^{13}C NMR (100 MHz, DMSO-d_6)

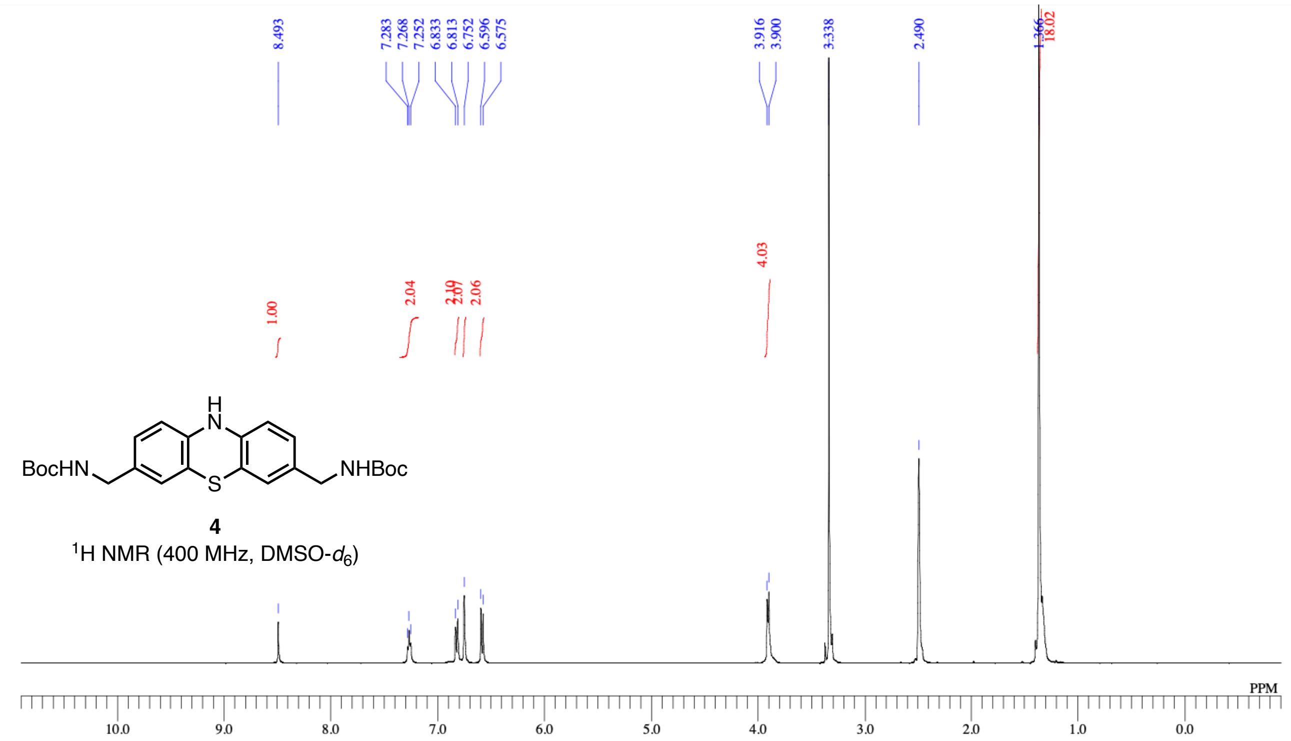
8.493
7.283
7.268
7.252
6.833
6.813
6.752
6.596
6.575
3.916
3.900
3.338
2.490
1.366
18.02
4.03
1.00
2.04
2.10
2.07
2.06
H
N
BocHN
NHBoc
S
4
^{1}H NMR (400 MHz, DMSO-d_6)
PPM
10.0
9.0
8.0
7.0
6.0
5.0
4.0
3.0
2.0
1.0
0.0

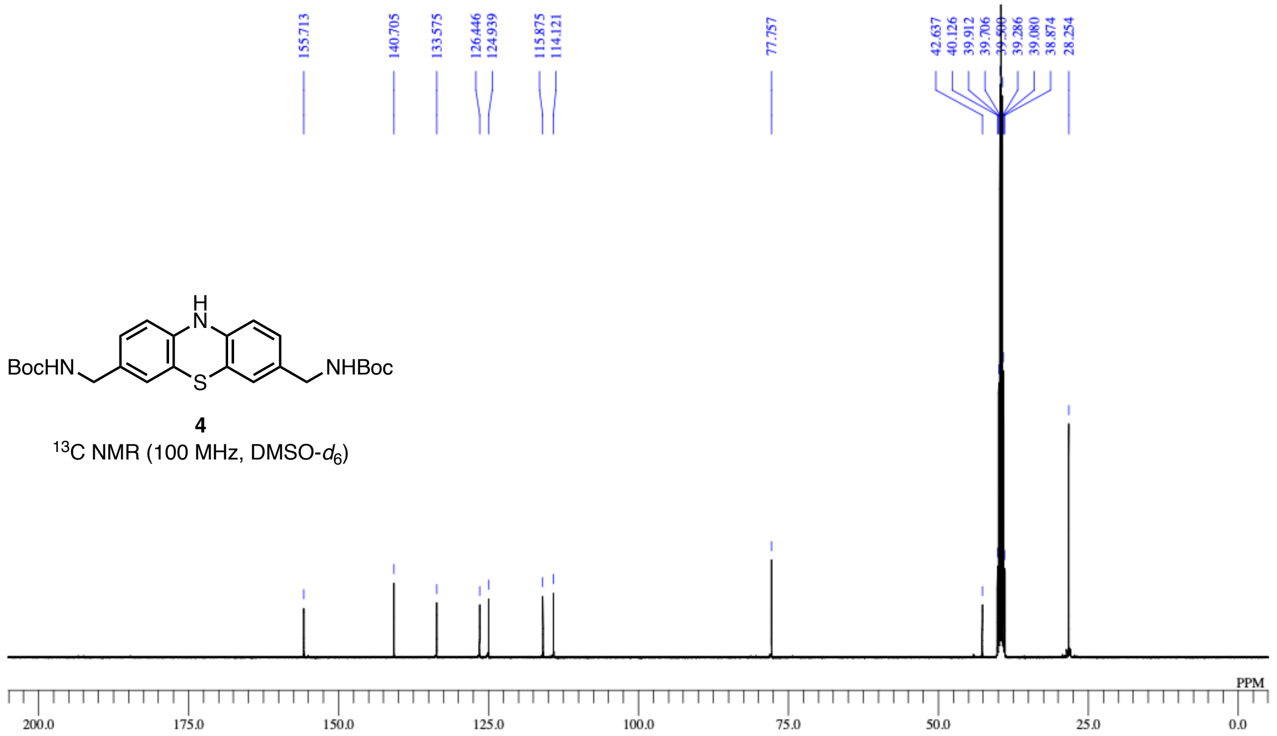
155.713
140.705
133.575
126.446
124.939
115.875
114.121
77.757
42.637
40.126
39.912
39.706
39.500
39.286
39.080
38.874
28.254
H
N
BocHN
NHBoc
S
4
^{13}C NMR (100 MHz, DMSO-d_6)
PPM
200.0
175.0
150.0
125.0
100.0
75.0
50.0
25.0
0.0

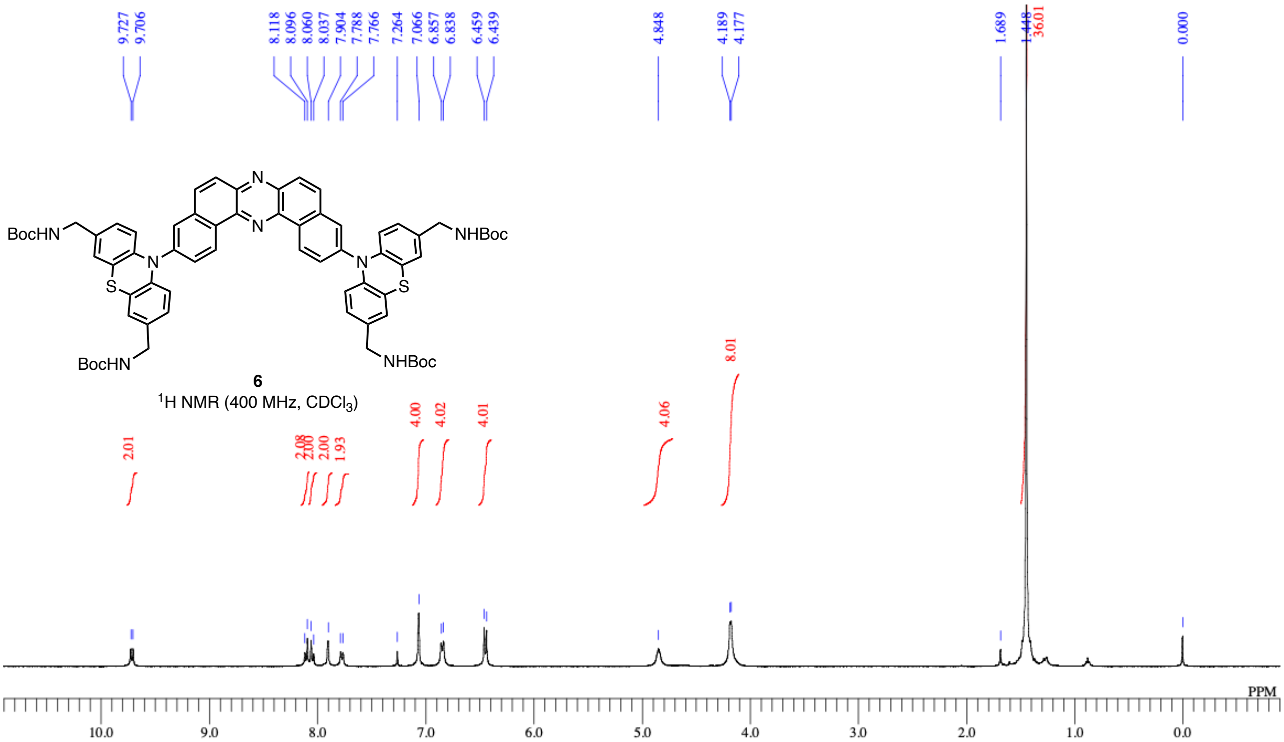

BocHN
NHBoc
BocHN
NHBoc
6
1H NMR (400 MHz, CDCl3)
9.727
9.706
8.118
8.096
8.060
8.037
7.904
7.788
7.766
7.264
7.066
6.857
6.838
6.459
6.439
4.848
4.189
4.177
1.689
1.448
0.000
2.01
2.08
2.00
2.00
1.93
4.00
4.02
4.01
4.06
8.01
36.01
PPM
10.0
9.0
8.0
7.0
6.0
5.0
4.0
3.0
2.0
1.0
0.0

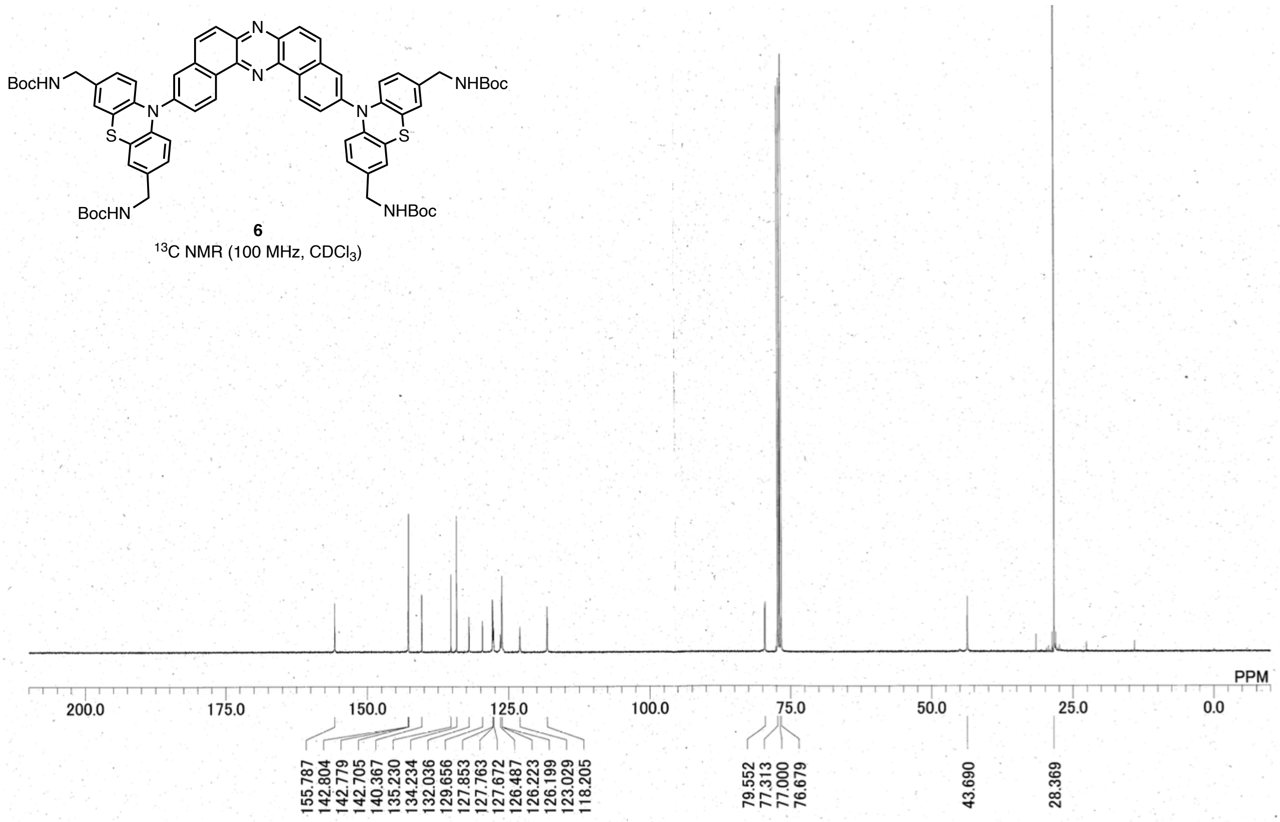

BocHN
NHBoc
BocHN
NHBoc
6
13C NMR (100 MHz, CDCl3)
PPM
200.0
175.0
150.0
125.0
100.0
75.0
50.0
25.0
0.0
155.787
142.804
142.779
142.705
140.367
135.230
134.234
132.036
129.656
127.853
127.763
127.672
126.487
126.223
126.199
123.029
118.205
79.552
77.313
77.000
76.679
43.690
28.369

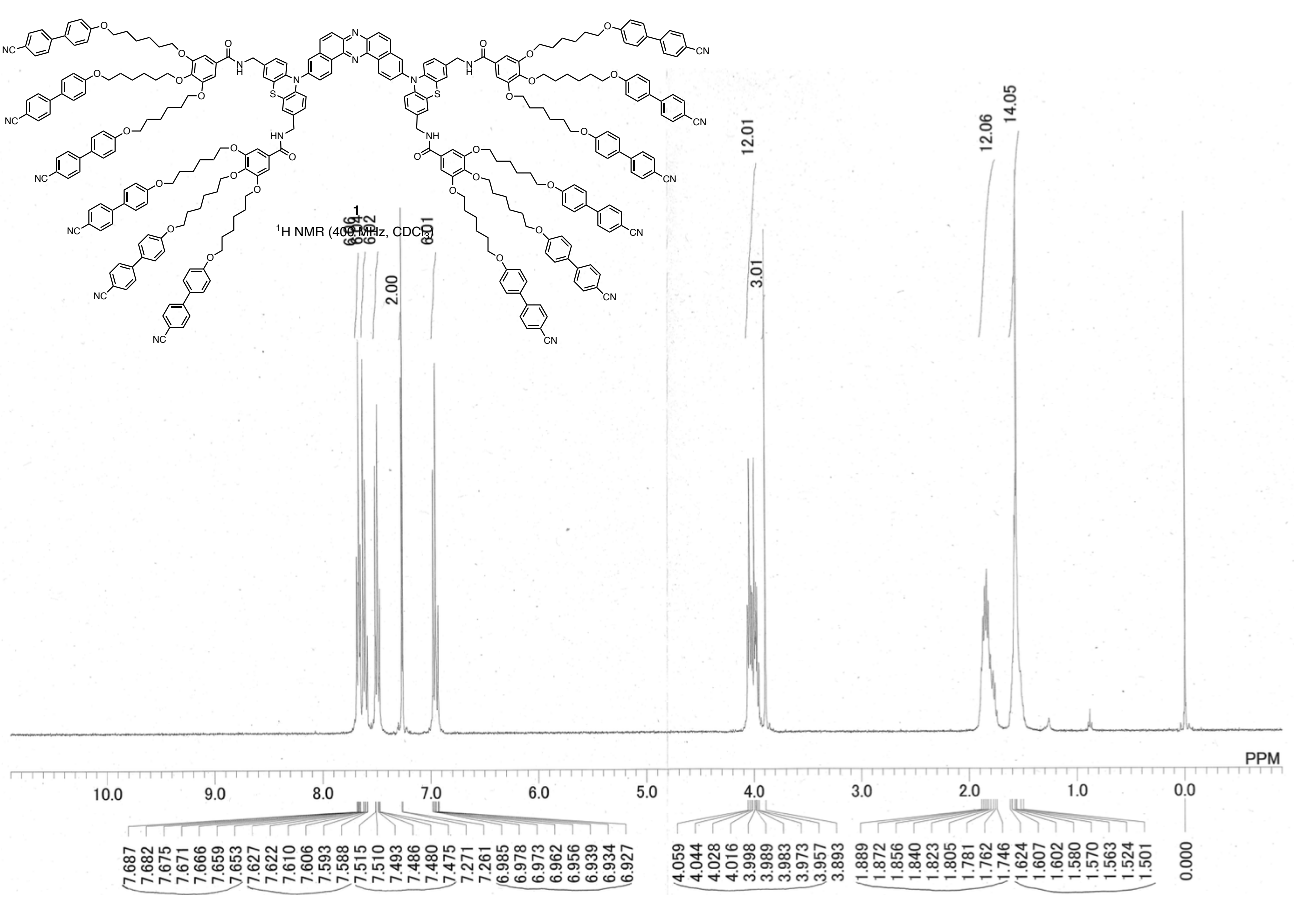

1
^{1}H NMR (400 MHz, CDCl$_3$)
12.01
3.01
12.06
14.05
2.00
PPM
10.0 9.0 8.0 7.0 6.0 5.0 4.0 3.0 2.0 1.0 0.0
7.687 7.682 7.675 7.671 7.666 7.659 7.653 7.627 7.622 7.610 7.606 7.593 7.588 7.515 7.510 7.493 7.486 7.480 7.475 7.271 7.261 6.985 6.978 6.973 6.962 6.956 6.939 6.934 6.927
4.059 4.044 4.028 4.016 3.998 3.989 3.983 3.973 3.957 3.893
1.889 1.872 1.856 1.840 1.823 1.805 1.781 1.762 1.746 1.624 1.607 1.602 1.580 1.570 1.563 1.524 1.501
0.000

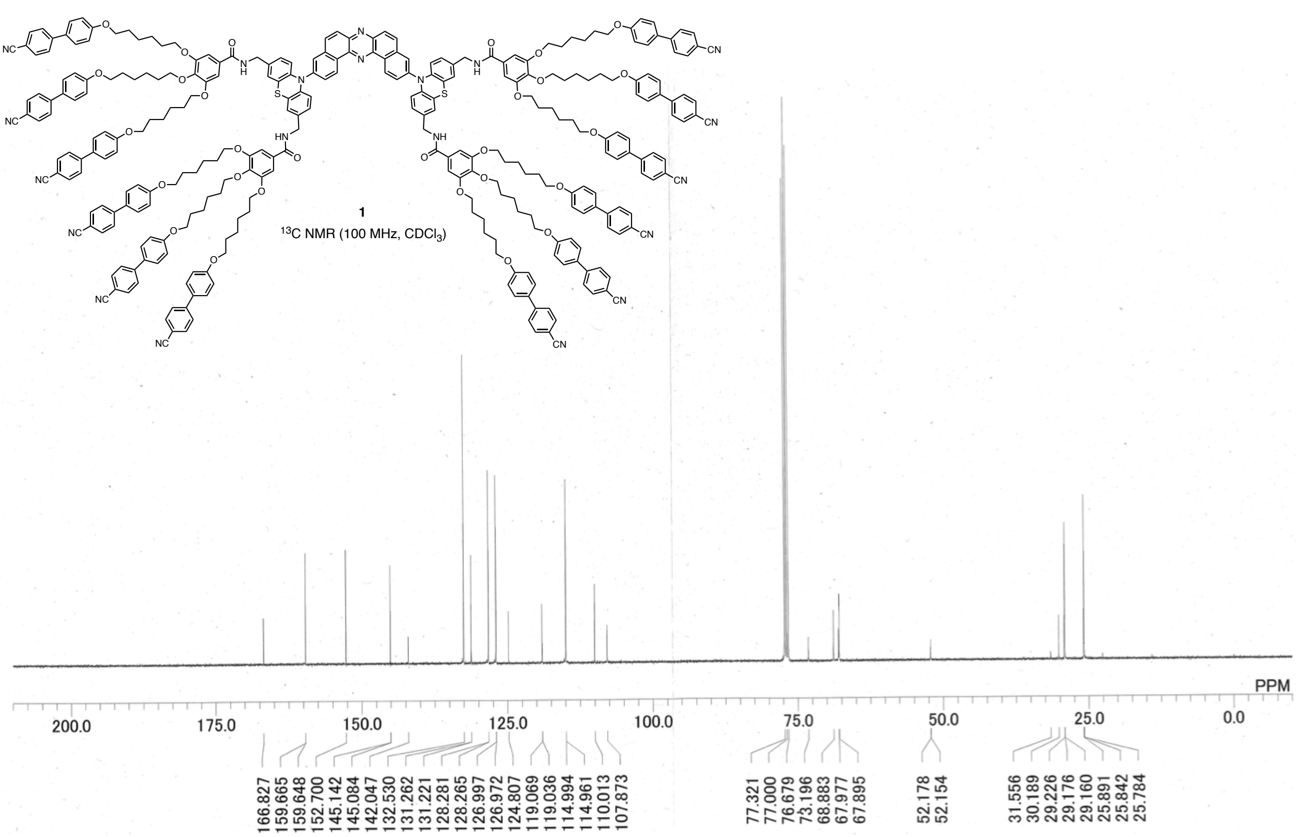

1
^{13}C NMR (100 MHz, CDCl$_3$)
PPM
200.0 175.0 150.0 125.0 100.0 75.0 50.0 25.0 0.0
166.827 159.665 159.648 152.700 145.142 145.084 142.047 132.530 131.262 131.221 128.281 128.265 126.997 126.972 124.807 119.069 119.036 114.994 114.961 110.013 107.873
77.321 77.000 76.679 73.196 68.883 67.977 67.895
52.178 52.154
31.556 30.189 29.226 29.176 29.160 25.891 25.842 25.784

References

[S1] Y. Takeda, M. Okazaki, S. Minakata, *Chem. Commun.* **2014**, *50*, 10291–10294.

[S2] A. Gandubert, M. Amela-Cortes, S. K. Nayak, C. Vicent, C. Mériadec, F. Artzner, S. Cordier, Y. Molard, *J. Mater. Chem. C* **2018**, *6*, 2556–2564.

[S3] M. Okazaki, Y. Takeda, P. Data, P. Pander, H. Higginbotham, A. P. Monkman, S. Minakata, *Chem. Sci*. **2017**, *8*, 2677–2686.

[S4] G. T. Williams, A. C. Sedgwick, S. Sen, L. Gwynne, J. E. Gardiner, J. T. Brewster, II, J. R. Hiscock, T. D. James, A. T. A. Jenkins, J. L. Sessler, *Chem. Commun.* **2020**, *56*, 5516–5519.

[S5] C. I. Bayly, P. Cieplak, W. Cornell, P. A. Kollman, *J. Phys. Chem.* **1993**, *97*, 10269–10280.

[S6] J. Wang, R. M. Wolf, J. W. Caldwell, P. A. Kollman, D. A. Case, *J. Comput. Chem.* **2004**, *25*, 1157–1174.

[S7] T. Enjou, S. Goto, Q. Liu, F. Ishiwari, A. Saeki, T. Uemtatsu, Y. Ikemoto, S. Watanabe, G. Matsuba, K. Ishibashi, G. Watanabe, S. Minakata, Y. Sagara, Y. Takeda, *Chem. Commun.* **2024**, *60*, 3653–3656.

[S8] G. Tiberio, L. Muccioli, R. Berardi, C. Zannoni, *ChemPhysChem* **2009**, *10*, 125–136.

[S9] K, Sano, Y. Itoh, F. Araoka, G. Watanabe, T. Hikima, T. Aida, *Science* **2019**, *363*, 161–165.

[S10] H. J. C. Berendsen, J. P. M. Postma, W. F. van Gunsteren, A. DiNola, J. R. Haak, *J. Chem. Phys.* **1984**, *81*, 3684–3690.

[S11] S. Nosé, *Mol. Phys.* **1984**, *52*, 255–268.

[S12] M. Parrinello, A. Rahman, *J. Appl. Phys.* **1981**, *52*, 7182–7190.

[S13] B. Hess, H. Bekker, H. J. C. Berendsen, J. G. E. M. Fraaije, *J. Comput. Chem.* **1997**, *18*, 1463–1472.

[S14] T. Darden, D. York, L. Pedersen, *J. Chem. Phys.* **1993**, *98*, 10089–10092.
